# KANN: estimation of genetic ancestry profiles by nearest neighbor regression

**DOI:** 10.1093/nar/gkag209

**Published:** 2026-03-12

**Authors:** Juha Riikonen, Sini Kerminen, Aki Havulinna, Matti Pirinen

**Affiliations:** Institute for Molecular Medicine Finland, Helsinki Institute of Life Science, University of Helsinki, 00014Helsinki, Finland; Institute for Molecular Medicine Finland, Helsinki Institute of Life Science, University of Helsinki, 00014Helsinki, Finland; Institute for Molecular Medicine Finland, Helsinki Institute of Life Science, University of Helsinki, 00014Helsinki, Finland; Department of Computing, University of Turku, 20014 Turku, Finland; Institute for Molecular Medicine Finland, Helsinki Institute of Life Science, University of Helsinki, 00014Helsinki, Finland; Department of Public Health, University of Helsinki, 00014Helsinki, Finland; Department of Mathematics and Statistics, University of Helsinki, 00014Helsinki, Finland

## Abstract

State-of-the-art methods for inferring individual-level genetic ancestry are based on statistical models for haplotype data. Unfortunately, these methods are computationally demanding, making them impractical for biobank-scale analyses. In this paper, we describe KANN, an efficient k-nearest neighbor regression method for individual-level ancestry estimation with respect to predefined source populations using only principal components of genetic structure. Contrary to the existing tools that can only use reference samples with discrete source population assignment, KANN enables the use of reference samples with continuous ancestry profiles across multiple source populations. We observe that KANN’s ancestry estimates agree well with the haplotype-based method SOURCEFIND when estimating ancestry profiles across up to 10 Finnish source populations on a dataset of 18 125 Finnish samples from THL Biobank. In the 1000 Genomes Project data containing globally diverse genetic backgrounds, KANN produces highly similar results to the ADMIXTURE software. Based on our results, KANN is a promising tool for ancestry estimation in large-scale genomic studies.

## Graphical Abstract

**Graphical Abstract:**
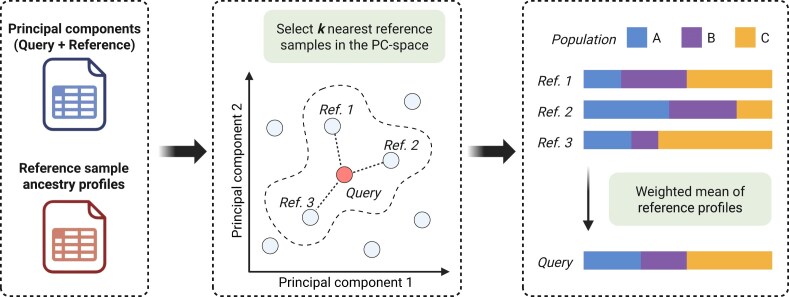

## Introduction

The genetic variation currently observed in human populations has been shaped by historical migrations and admixture events. Understanding and quantifying this genetic variation is essential in population genetics and genomic medicine. For example, the performance and reliability of polygenic risk scores (PRS), which have been successfully used for quantifying the genome-wide contribution to complex diseases and traits [1], depend heavily on the genetic ancestry of individuals [2, 3]. Therefore, the detailed characterization of individual-level ancestry is essential for selecting the most suitable ancestry-matched PRS model for each individual. In addition, the general public has shown great interest in their own genetic ancestry, as millions of individuals have already taken a direct-to-consumer genetic test provided by companies with affordable services and easily accessible online platforms [4]. Thus, there is a clear need to scale accurate ancestry estimation methods to biobank-scale datasets.

Various computational tools have been developed for the purpose of genetic ancestry inference. SOURCEFIND [5] and RFMix [6] are widely used methods that utilize individual-level haplotype information. Such haplotype-based methods are able to distinguish fine-scale genetic structure but suffer from slow runtime. Compared to the haplotype-level models, methods that work with individual-level genotype data (e.g. iAdmix [7] and ADMIXTURE [8]) can improve on runtime, but still they remain computationally heavy for large samples.

A recent approach called Rye [9] introduced the idea of basing genetic ancestry inference on individuals’ scores on principal components of genetic structure without a need to access variant-level data. This enabled more efficient computation, making Rye applicable to biobank-scale inference. Rye was shown to run over 50 times faster than ADMIXTURE when applied to a dataset of 488 221 UK Biobank participants, while the accuracy remained comparable to the existing methods (RFMix, iAdmix, and ADMIXTURE) [9]. Recently, PBWTpaint [10] introduced haplotype components that could potentially be used as a haplotype-based alternative for principal components in ancestry inference.

As an input, Rye requires a set of reference individuals that are each assigned to represent a single ancestral source population. This can be a sufficient approach when only discrete population information is available, and the ancestral populations are carefully defined to ensure they are relatively homogeneous and minimally admixed with one another. However, the discrete assignment likely oversimplifies the complexity of population structure we observe in modern human populations. A more flexible approach would be to use a continuous representation of ancestry, where each reference individual is characterized by an ancestry profile describing the proportion of genetic ancestry inherited from each of the source populations. This framework would allow for a more detailed depiction of genetic ancestry and has the potential to increase the precision of ancestry inference, especially in highly admixed populations.

In this work, we develop a new method for $k$-nearest neighbor regression for ancestry estimation (KANN), which works in the space of principal or haplotype components of genetic structure. Importantly, KANN can utilize admixed reference individuals who have proportions of genetic ancestry inherited from multiple source populations. Such ancestry profiles for reference samples could be obtained using existing methods, including SOURCEFIND and ADMIXTURE. As a special case, KANN is applicable to the conventional ancestry format where each reference sample is assigned to exactly one population, allowing a direct comparison with Rye.

We apply KANN to a dataset from THL biobank that contains fine-scale ancestry profiles with respect to 10 Finnish subpopulations made by SOURCEFIND for 18 125 individuals. We compare the results from KANN and Rye to SOURCEFIND, and we interpret the observed differences with respect to the known genetic and geographical relationships of the Finnish subpopulations. KANN is benchmarked with both principal components obtained via PLINK 2.0 and haplotype components obtained via PBWTpaint. In addition, we apply KANN to genetically diverse data of 2504 global reference samples from the 1000 Genomes Project (1KGP). There, KANN is compared to ancestry fractions previously inferred using the ADMIXTURE software.

## Materials and methods

### Software availability

KANN is available as a software package in R-language at https://github.com/riikonenj/KANN.

### Finnish data

We used data from the biobank of the Finnish Institute for Health and Welfare (THL), consisting of 16 962 023 variants measured on 51 852 individuals, together with the birth locations up to a municipality-level precision. The data originated from Finnish cohort studies: FINRISK (*n* = 30 867), GeneRISK (*n* = 7255), FinHealth 2017 (*n* = 6155), and Health 2000 (*n* = 7575). All individuals included in this study had given written consent.

### PCA on the Finnish data

As KANN operates on the principal components (PCs) of the genetic structure [11, 12], it is crucial to start from a high-quality principal component analysis (PCA) achieved through stringent variant and sample quality control steps.

#### Variants

Variant quality control was performed using PLINK 2.0 software [13]. We considered only biallelic variants found in the autosomal genome. We followed a widely used practice of using common variants in genotype PCA by excluding variants with minor allele frequency (MAF) $< $ 5% [12, 14]. In addition, we excluded variants with Hardy–Weinberg $P$-value $< $ 1e-6 to avoid badly genotyped or multiallelic variants and an imputation information score $< $ 95% to avoid variants where imputation was less certain. High amounts of linkage-disequilibrium (LD) can distort PCA-based inference by describing local LD structure instead of genome-wide population structure [11, 12]. To consider only nearly independent variants, we removed known regions of long-range LD [15], and pruned the remaining variants so that the squared pairwise correlation $r^2 \le 0.2$. After filtering, 94 843 variants remained.

#### Samples

Starting from 51 852 samples, we considered only those whose municipality of birth was in Finland or in the eastern regions ceded to the Soviet Union during the Second World War. We estimated the sample heterozygosity using a method of moments F-statistic and excluded samples whose F-statistic deviated $> 4$ standard deviations (SD) from the mean. The exclusion of related samples prior to PCA is a common approach to ensure that the PCs reflect population-level structure instead of family structures [11, 12]. For this purpose, we used the KING kinship coefficient threshold $\phi > 0.0442$ [16] to prune out the relatives starting from the third degree. After filtering, 38 113 unrelated samples remained.

#### PCA

Following the variant and sample filtering, we applied PCA on the genotypes of the 38 113 unrelated samples. The PCA was run using PLINK 2.0 software with the approximation modifier (--pca approx). We extracted the first 20 principal components (PCs) and the corresponding eigenvalues. An additional 9837 non-monozygotic samples with excess kinship or homozygosity were afterwards projected on the PCs, resulting in a total of 47 950 samples with the PC values. Finally, the PCs were standardized to have a zero mean and unit variance.

### SOURCEFIND ancestry profiles

A fine-scale genetic population structure in Finland was previously determined [17] using the FINRISK study cohorts included in our data. In short, a subset of 2741 carefully chosen reference individuals was allocated to three sets of genetically and geographically well-defined reference groups in an iterative process utilizing the ChromoPainter and FineSTRUCTURE software [18]. The reference sets characterize the fine-scale genetic ancestry in Finland using two (refset 2, *n* = 1472), six (refset 6, *n* = 1026), and ten (refset 10, *n* = 1236) reference groups per set. SOURCEFIND [5] was further used to estimate two-, six-, and ten-dimensional ancestry profiles for a total of 18 463 samples, including the reference samples themselves. SOURCEFIND was run with 50 000 burn-in iterations, 200 000 sample iterations, and results were recorded after every 5000 iterations. When estimating ancestry for each of the reference group samples, the sample itself was left out of its reference group.

**Figure 1.**
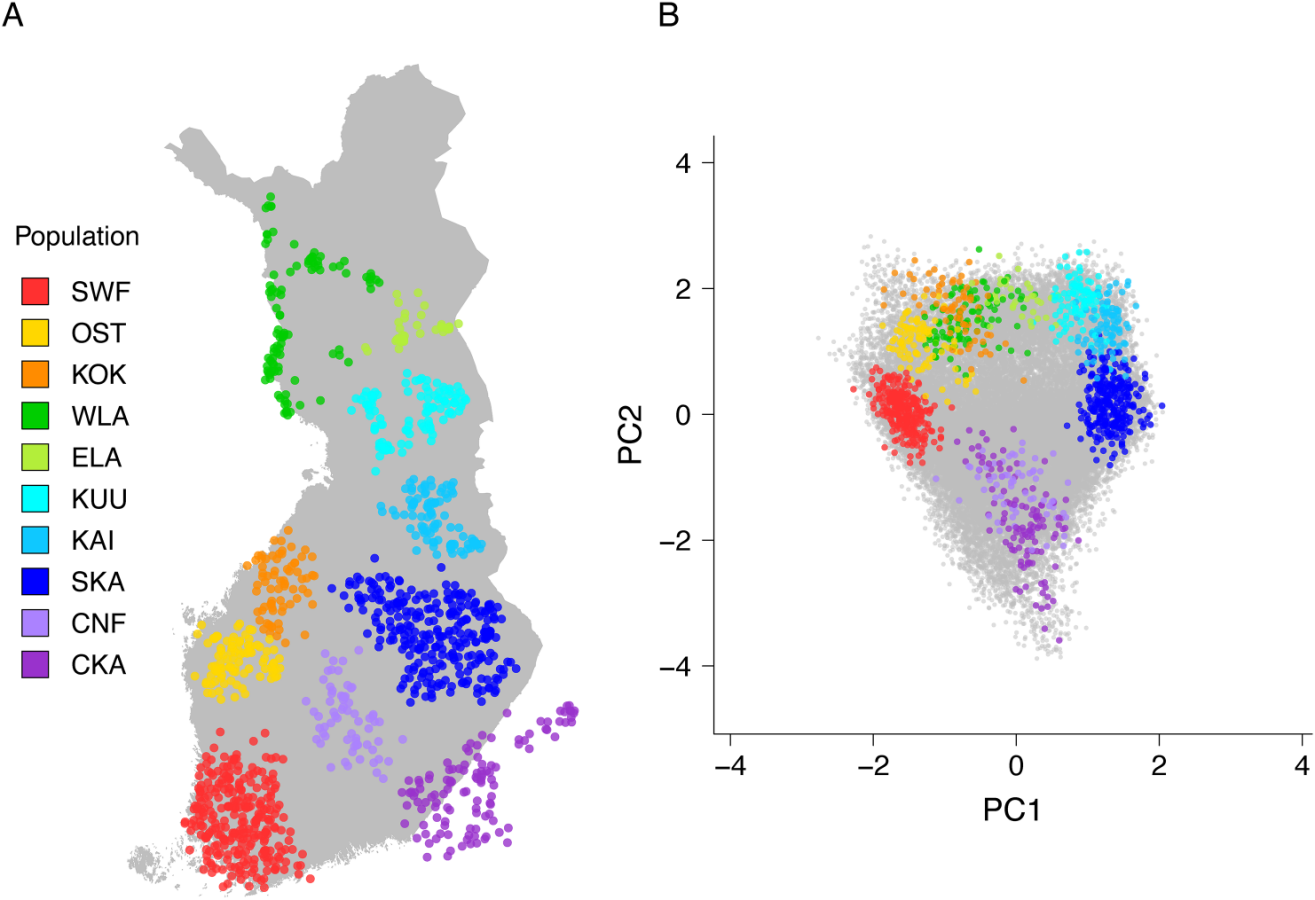
**A**) Map of Finland with the geographical locations of the reference samples (*n* = 1214) allocated to 10 Finnish source populations. The points depict the mean coordinates of the parents’ municipalities of birth. (**B**) The same individuals are highlighted on the first two principal components. The samples not included in the reference groups are depicted in grey colour. SWF: Southwestern Finland, OST: Ostrobothnia, KOK: Kokkola, WLA: West Lapland, ELA: East Lapland, KUU: Kuusamo, KAI: Kainuu, SKA: Savo-Karelia, CNF: Central Finland, CKA: Ceded Karelia.

We had both our own PC coordinates and the SOURCEFIND profiles from [17] available for 18 125 FINRISK samples. Out of these samples, 1798 were among the reference samples (1443, 1006, and 1214 had a discrete population assignment with respect to 2, 6, and 10 source populations, respectively). The data quality control and pre-processing steps are described in Supplementary Fig. S1. The geographical locations of the 1214 samples forming the 10 source populations are depicted in Fig. 1A and B shows the same samples on the first two PCs. Supplementary Figs S2–S4 show the same information for the first 10 PCs and for 2, 6, and 10 populations, respectively.

### KANN algorithm

#### k-nearest neighbor regression

Ancestry estimation with KANN leverages the known ancestry profiles of $n_R$ reference samples, each having a proportion of their genetic ancestry assigned to $S$ different source populations. We represent the ancestry profile of the reference sample $j \le n_{R}$ as


\begin{eqnarray*}
\pmb {r}_{j} = ( r_{j,1}, r_{j,2}, \dots , r_{j,S} ),
\end{eqnarray*}

where $r_{j,s}$ describes the ancestry proportion of sample $j$ with respect to the source population $s \le S$. Leveraging the known ancestry information of the reference samples, the aim is to estimate, for each query sample $i \le n_{Q}$, a corresponding ancestry profile:


\begin{eqnarray*}
\pmb {q}_{i} = ( q_{i,1}, q_{i,2}, \dots , q_{i,S} ) .
\end{eqnarray*}

To apply k-nearest neighbor regression for ancestry estimation, we define the distance $d(i,j) \ge 0$ between the query sample $i$ and the reference sample $j$ as their Euclidean distance in the $M$-dimensional PC-space, where the squared distance along each component $m$ is weighted with the component’s eigenvalue $\lambda _{m}$:


\begin{eqnarray*}
d(i,j) = \sqrt{ \sum _{m=1}^{M} \lambda _{m} (x_{i,m} - x_{j,m})^2 } \quad ,
\end{eqnarray*}

where $x_{i,m}$ and $x_{j,m}$ are, respectively, the scores of the query sample $i$ and the reference sample $j$ on the principal component $m$. The $M$ eigenvalues $\lambda _m$ are normalized to sum to one. The time complexity of the distance matrix computation is $O(n_Q n_R M)$.

Given $k \le n_{R}$, we denote for each query sample $i$ the set of indices of its $k$ nearest reference samples by $\mathbf {J}^{(k)}_{i}$. The ancestry component $q_{i,s}$ is computed as the arithmetic mean over the ancestry components of the $k$ nearest reference samples:


\begin{eqnarray*}
q_{i,s} = \frac{1}{k} \sum _{j \in \mathbf {J}^{(k)}_{i}} r_{j,s} \quad .
\end{eqnarray*}

The time complexity of the ancestry estimation is dominated by sorting the distance matrix and is of the order $O\left(n_Q n_R \mathrm{log}(n_R)\right)$. Therefore, the runtime of the KANN algorithm is of the order $O\left(n_Q n_R( \mathrm{log}(n_R)+M)\right)$.

#### Inverse distance weighting

In case there is a high variability among the distances between the query sample and its $k$ nearest reference samples, it is plausible that the reference samples closest to the query sample are more descriptive of its ancestry than those farther away. With this in mind, we define, for a given exponent $p \ge 0$, the weight $w(i,j) = d(i,j)^{-p}$ of reference sample $j$ on query sample $i$, based on their inverse pairwise distance raised to the power $p$. The ancestry components are then estimated as


\begin{eqnarray*}
q_{i,s} = \frac{ \sum _{j \in \mathbf {J}^{(k)}_{i}} w_{i,j} \: r_{j, s} }{ \sum _{j \in \mathbf {J}^{(k)}_{i}} w_{i,j} } \quad .
\end{eqnarray*}

To avoid extremely large weights, KANN can be provided with a minimum distance threshold $\varepsilon$ that is substituted for the distances $< \varepsilon$. In our applications, we set $\varepsilon = 0.1$.

### Total variation distance

Ideally, for the same input data, KANN would estimate similar ancestry profiles as the current state-of-the-art haplotype-based ancestry estimation methods. To assess the performance of KANN, we compare our estimated sample profiles with the SOURCEFIND profiles for the same samples. As a distance measure between two $S$-dimensional ancestry profiles $\pmb {q}^{(1)}_i$ and $\pmb {q}^{(2)}_i$ we use the total variation distance (TVD), defined as


\begin{eqnarray*}
\mathrm{TVD}\left( \pmb {q}^{(1)}_{i}, \pmb {q}^{(2)}_{i} \right) = \frac{1}{2} \sum _{s=1}^{S} \left| q^{(1)}_{i,s} - q^{(2)}_{i,s} \right|.
\end{eqnarray*}

### Model building and test datasets

From the 18 125 samples, we extracted a test set of 1000 samples that did not overlap with the 1798 samples used to represent the Finnish source populations in the SOURCEFIND analysis. To ensure that all 10 source populations were represented in the test set, for each population, we randomly selected five such samples that had $\ge$75% of their ancestry assigned to the population by SOURCEFIND. This property of the test set also holds with respect to either two or six source populations. The remaining 950 samples in the test set were selected randomly. The 17 125 samples not included in the test set are referred to as the model-building dataset.

### Parameter optimization in the Finnish data

We optimized parameters $k$ and $p$ within the model-building dataset by minimizing TVD between our estimated ancestry profiles and those available for the same samples from SOURCEFIND. Hereafter, unless otherwise specified, we use ‘TVD’ to refer to the total variation distance between the SOURCEFIND profile and an ancestry profile estimated using some other method. In addition to TVD, we computed the mean of the Pearson correlation coefficients between the estimates of each ancestry component in the query profiles and the corresponding SOURCEFIND profiles.

This optimization process was repeated separately for 2, 6, and 10 source populations, and for three different ways to define the reference profiles. To assess how the number of top principal components utilized by KANN affects estimation accuracy, we repeated the optimization process while varying the number of PCs from 1 to 20. Apart from this assessment, subsequent analyses were conducted using ancestry profiles generated from the top 10 PCs, unless otherwise specified. In each setting, the model-building dataset was divided into reference samples and query samples. Instead of an exhaustive search through all possible parameter values, we applied the optimization in a restricted parameter space. For the parameter $p$, we ran KANN for integer values from 0 to 10. When $p = 0$, an equal weight is assigned to all pairwise distances, corresponding to the case where no distance weighting is applied. Depending on the setting, we ran the algorithm with different increments of $k$ starting from 1. The three scenarios for defining the reference profiles are as follows.

#### DISCRETE

In the DISCRETE scenario, the reference samples are those with a discrete assignment to a single population, i.e., 100% of the ancestry of a reference sample is assigned to one source population. This approach enables a meaningful comparison between KANN and such ancestry inference tools that require discrete reference profiles. In our data, we have discrete population assignments for the samples that were used as the reference samples in the original SOURCEFIND analysis. For parameter $k$, we examine values 1, 10, 25, 50, 100, 250, 500, 1000, and $n_R$, where $n_R$ is the maximum number of reference samples available in each set. This equals 1443 samples (2 populations), 1006 samples (6 populations), and 1214 samples (10 populations).

#### CONTINUOUS

In the CONTINUOUS scenario, we use the same reference set as in the DISCRETE scenario, namely 1443 samples (2 populations), 1006 samples (6 populations), and 1214 samples (10 populations), and the same range of values for the parameter $k$. The difference here is that we utilize the continuous ancestry profiles estimated by SOURCEFIND, where the individual’s profile consists of proportions of ancestry assigned to all $S$ source populations. This way, we can demonstrate KANN’s ability to utilize reference samples with continuous ancestry profiles.

#### CONTINUOUSALL

The CONTINUOUSALL scenario is an extension of the CONTINUOUS scenario, where we again utilize information on continuous ancestry profiles, but use all available samples from the model-building dataset as our reference set. For parameter optimization, we use cross-validation. First, the samples are randomly assigned to 10 cross-validation folds, each containing approximately 10% of the samples. Each fold in turn is considered as the query samples, and the remaining folds are the reference samples. Compared to the DISCRETE and CONTINUOUS scenarios, we now have a larger number of reference samples available. We extend the parameter space for $k$ to cover values 1, 3, 5, 10, 25, 50, 100, 250, 1000, 5000, 10 000, and 15 000.

The optimal pair of parameters in the DISCRETE and CONTINUOUS scenarios is the one that minimizes the mean TVD in the query sample set. In the CONTINUOUSALL scenario, the optimal pair is the one that minimizes the mean TVD over the combined set of query profiles from the 10 cross-validation folds. After the parameter optimization, we estimated ancestries for the test set of 1000 samples using KANN with different numbers of source populations and for the three scenarios of reference profiles. In each case, the algorithm was run with 10 PCs and the parameter pair found during the optimization process.

### Comparison with Rye

We used the Finnish data to compare the accuracy and runtime of KANN to Rye, a recent software tool for genetic ancestry inference based on principal components applicable at a biobank scale [9]. Rye uses Markov chain Monte Carlo optimization to find vectors in the PC-space to represent each of the ancestral source populations. Genetic ancestry profiles for the query samples are then estimated by regressing their PC vectors on those of the source populations using non-negative least squares regression. Rye was shown to be comparable in accuracy with RFmix [6], ADMIXTURE [8], and iAdmix [7], and outperforms them with respect to runtime [9].

We used Rye to estimate ancestry profiles for the 1000 test set samples with respect to 2, 6, and 10 Finnish source populations, and compared the estimated profiles with SOURCEFIND using TVD. In all cases, we used 10 PCs and the default parameters. Rye, like many ancestry inference methods, uses reference samples allocated to a single source population. Thus, we used the same reference sample set for Rye as we used in our DISCRETE scenario, because those are the only samples in our data with a discrete assignment to source populations. This enabled a meaningful comparison between Rye and KANN, since identical input data was used with both methods.

Further, we compared the runtime between Rye and KANN in estimating the test set profiles with respect to 10 source populations. Both methods used the same 1214 reference samples with discrete ancestry information and the first 10 PCs. KANN’s runtime includes the pairwise distance matrix computation between the reference and test samples. KANN was run with parameters $k=n_R-1=1213$ and $p=3$. (As KANN is exceptionally fast in the special case $k = n_R$, where the nearest neighbor search can be skipped, for this runtime comparison, we ran KANN using $k = n_R - 1$ instead of the optimized value $k=n_R$.) Taking into account the possible variation in runtimes, we report the median and range of runtimes from 10 separate runs for both algorithms. Runtime was measured using an Intel Xeon Gold 6148 CPU and 173GB of RAM, with KANN first running on a single core and then on four cores, while Rye utilized up to four cores.

### Interpreting the TVD values

TVD between an individual’s profile estimated by KANN and SOURCEFIND tells which proportion of ancestry KANN has assigned to different source populations than SOURCEFIND. Thus, small TVD values between KANN and SOURCEFIND indicate that KANN can provide similar accuracy to a state-of-the-art haplotype-based ancestry estimation method. As TVD adds up the absolute differences over all ancestry components, it does not directly tell for which population pairs the difference occurred. We studied further the magnitude of the differences for individual source populations, and determined whether some pairs of the source populations get mixed up by the two methods more often than others. These analyses were conducted on the test set where the ancestry profiles were estimated using the parameter values optimized in the model-building phase.

First, we looked at the marginal differences $\mathrm{KANN} - \mathrm{SOURCEFIND}$ between the individual ancestry components in the test set. Furthermore, we studied how the marginal differences of the other populations are distributed among the samples that have a high marginal difference in one population. In turn, we took each source population as our target population and identified the test set samples with a marginal difference $\ge 0.05$ for that population. The individuals with a high marginal difference in the target population were divided into two groups based on the sign of the difference (positive or negative). A positive difference implies that, compared to SOURCEFIND, KANN had overestimated the ancestry component of the population. Correspondingly, a negative difference implies that KANN had underestimated the component. For the samples in each group, we calculated the arithmetic means of the marginal differences with respect to all other populations. We were interested in which populations the marginal difference was in the opposite direction than in our target population, as that suggests that those populations and our target population had been mixed up between KANN and SOURCEFIND. We normalized the absolute values of the mean differences that were in the opposite direction compared to the target population’s difference, and we ignored the possible differences with the same sign as the difference of the target population.

### Haplotype component analysis (HCA)

As input data to KANN, we may use haplotype components obtained via the PBWTpaint software [10] instead of the principal components of genetic structure. PBWTpaint is an extension of the Positional Burrows-Wheeler Transform (PBWT) algorithm, which is used to identify long matches between haplotypes. PBWTpaint can be used to conduct all-vs-all chromosome painting on a dataset and represent the sample relationships in a sparse matrix of the length of shared haplotypes. Following the pipeline of Yang *et al.* [10], we ran PBWTpaint on the same dataset of 38 113 samples that we used for the PCA of the Finnish data. While PCA works with nearly independent variants, PBWTpaint can utilize haplotype information carried by variants in high LD. Therefore, we provided PBWTpaint with 5 637 276 variants, satisfying the same quality control filters as the PCA variants of the Finnish dataset, except that no LD-pruning was applied. We extracted the first 20 haplotype components (HCs) and their singular values, and standardized each HC to have zero mean and unit variance. We reran the optimization process for the parameter pair $(k,p)$ by replacing PCs with HCs.

As 2117 Finnish samples with SOURCEFIND profiles were not included in the PCA dataset, but were only later projected to the PCs, they are missing the HCs. For this reason, the HCA model-building dataset (*n* = 15 136) is a subset of the PCA model-building dataset. The number of samples belonging to the SOURCEFIND reference sets in the HCA model-building dataset is 1266 samples (2 populations), 882 samples (6 populations), and 1085 samples (10 populations). Because of the smaller size of the HCA model building dataset, we restricted the parameter space for $k$ in the DISCRETE and CONTINUOUS scenarios to 1, 10, 25, 50, 100, 250, 500, and $n_R$, where $n_R$ was the maximum number of reference samples available in each set. Similarly, we restricted $k$ in the CONTINUOUSALL scenario to 1, 3, 5, 10, 25, 50, 100, 250, 1000, 5000, 10 000, and 13 000.

To assess the overall performance of KANN while using HCs in ancestry estimation, we compared the mean TVDs obtained in the query dataset using HCs to those obtained using PCs at the optimal parameter values of each analysis. We also studied how the optimized mean TVD varies with the number of top HCs provided to KANN.

### 1000 Genomes Project ADMIXTURE analysis

To benchmark the performance of KANN in a genetically diverse context, we used genotype data from the 1000 Genomes Project (1KGP; phase 3 data, 2504 samples) [19] together with ancestry profiles previously inferred by the ADMIXTURE software using 193 634 variants. For this purpose, we performed quality control and PCA using PLINK 2.0 on the dataset that we downloaded from the 1KGP website.

The data had already been filtered for the ADMIXTURE analysis to include only biallelic SNPs with MAF $> $ 5% that are at least 2Kb apart from each other. We further excluded regions of extended linkage disequilibrium [15]. Additional LD pruning was applied with the threshold for squared pairwise correlation $r^2 \le 0.2$, resulting in 81 573 variants. Related pairs (KING kinship coefficient $\phi > 0.0442$) were removed, leaving 2465 unrelated samples for PCA. The first 20 PCs were extracted, and the remaining 39 related individuals were subsequently projected onto the PC space. All PCs were scaled to have a zero mean and unit variance.

We compared KANN estimates with ADMIXTURE ancestry proportions inferred at the level of 5 source populations. An examination of the original ADMIXTURE results in 1KGP data showed that out of the five source populations, POP1 and POP2 represented African, POP3 European, POP4 South Asian, and POP5 East Asian genetic ancestry. As our target individuals, we selected the Colombian in Medellin (CLM, n = 94). We randomly split them into a query sample set for parameter optimization and a test set (*n* = 47 in both sets). The remaining 2410 individuals were used as the reference set with known continuous ADMIXTURE profiles. KANN parameters were optimized in the query sample set using the first 10 PCs, and the parameter grid for $p$ from 0 to 10, and $k$ at 1, 10, 25, 50, 100, 250, 500, 1000, 1500, 2000, and $n_R$ = 2410. For each parameter pair, query set profiles were estimated by KANN from the 5-population ADMIXTURE profiles of the reference samples. The optimal parameter pair minimizing the mean TVD between KANN and ADMIXTURE in the query set was then applied to estimate ancestry profiles for the independent test set.

## Results

### Optimal parameters in the Finnish data

The parameter optimization yields a pair of parameters $(k, p)$ that minimizes the mean TVD in the query dataset (Table 1). Unless otherwise specified, we report here the results from the KANN parameter optimization with 10 PCs. The smallest optimal $p$ encountered was 3, indicating that the ancestry estimation benefits from the distance weighting. In the DISCRETE and CONTINUOUS scenarios, the optimal number of nearest reference samples $k$ was always relatively close to $n_R$, the total number of reference samples. When a larger reference sample set was used in the CONTINUOUSALL scenario, the optimal $k$ was much smaller ($k = 25$ or $k = 50$).

**Table 1.** Mean TVD in the test set with 95% confidence intervals for Rye and three versions of KANN (both run with 10 PCs) for different numbers of source populations $S$. Optimal values of $p$ and $k$ are shown for KANN

RYE			
$S$			Mean TVD
2			0.082 (0.077, 0.086)
6			0.177 (0.170, 0.184)
10			0.244 (0.237, 0.252)
**KANN-DISCRETE**			
** $S$ **	**p**	**k**	**Mean TVD**
2	3	1443	0.074 (0.07, 0.079)
6	3	1000	0.138 (0.129, 0.146)
10	3	1214	0.186 (0.175, 0.198)
**KANN-CONTINUOUS**			
** $S$ **	**p**	**k**	**Mean TVD**
2	4	1000	0.061 (0.058, 0.065)
6	5	1000	0.119 (0.111, 0.126)
10	5	1214	0.148 (0.139, 0.157)
**KANN-CONTINUOUSALL**			
** $S$ **	**p**	**k**	**Mean TVD**
2	4	50	0.036 (0.034, 0.038)
6	3	25	0.069 (0.065, 0.074)
10	3	25	0.112 (0.105, 0.119)

Supplementary Fig. S5 shows the mean TVD, and Supplementary Fig. S6 shows the mean correlation observed in the query data across the parameter values. For the parameter $p$, values are shown from 0 to 6, since we did not observe any optimal parameter pairs with larger values of $p$ during the optimization process when using the first 10 PCs. Regardless of the scenario, the worst results with respect to TVD were observed when the inverse distance weighting was not applied and $k$ equals to its maximum possible value $n_R$. This is not surprising, as such a choice corresponds to estimating the same profile for all query samples. The weighting parameter $p$ had less impact on the mean TVD and correlation when $k$ is small. For example, in the KANN-CONTINUOUSALL scenario, where the optimal $k\le 50$, changes in $p$ showed less than a 0.001 difference in the mean TVD. However, in the same scenario, with a large $k = 15 \, 000$, we saw up to a 0.35 difference in the mean TVD between values $p = 0$ and $p = 5$.

Supplementary Fig. S7 reports the minimum mean TVD found in the query sample set across all considered parameter pairs $(k,p)$ when KANN optimization is run in the PCA model building dataset with a different number of top principal components (in gray). The minimum mean TVD declines strongly after the inclusion of the second and third components. In most scenarios, the TVD has stagnated after approximately 8 PCs. Including more than 10 PCs does not seem to noticeably improve the estimation accuracy.

### Ancestry estimation accuracy and runtime

**Figure 2.**
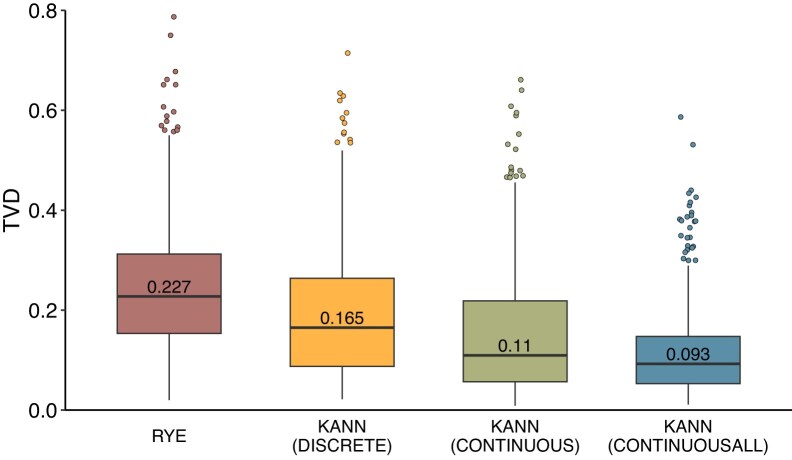
Box plots showing the TVD distribution of the test sample profiles with respect to 10 populations. Profiles are estimated using Rye and the three versions of KANN. The profiles in each version of KANN have been estimated using the optimal parameter pair from Table 1. The median TVD is shown on each box plot.

Fig. 2 shows the TVD distributions of the test set profiles estimated using KANN and Rye with respect to 10 Finnish source populations. (Supplementary Figs S8 and S9 show the distributions with respect to 2 and 6 populations, respectively.) The box plots show a similar trend with respect to the median TVD, as Table 1 shows with respect to the mean TVD. As expected, TVD increases with the number of source populations for every method. In all cases, Rye shows the largest TVD. This is followed by KANN-DISCRETE that provides a meaningful comparison with Rye, since there KANN is run with an identical reference sample set and the same discrete ancestry information as Rye. Incorporating continuous ancestry information with KANN-CONTINUOUS decreases TVD compared to using the discrete information. Finally, extending the reference sample set to cover the whole model-building dataset with KANN-CONTINUOUSALL shows the smallest TVD.

In the test set, KANN and SOURCEFIND ancestry estimates for each individual source population had a correlation $\rho = 0.988$ with 2 populations (Supplementary Fig. S10), $\rho \ge 0.963$ with 6 populations (Supplementary Fig. S11), and $\rho \ge 0.859$ with all 10 populations (Supplementary Fig. S12). Here, the KANN profiles had been estimated using ancestry information and reference samples from the KANN-CONTINUOUSALL scenario, and the optimal parameters corresponding to the number of source populations from Table 1.

For the runtime comparison, Rye and KANN were run on an identical set of 1214 reference samples and the 1000 test set samples as query individuals. The median runtime for KANN over 10 runs was 9.6 s (min = 9.4 s, max = 9.9 s) using one core and decreased to 4.0 s (min = 3.9 s, max = 4.7 s) using four cores. Rye took considerably longer, with a median runtime of 616 s (min = 541 s, max = 679 s) using four cores. We confirmed that the runtime of KANN was linear in the number of query samples, both with one and with four cores.

### Differences between KANN and SOURCEFIND

**Figure 3.**
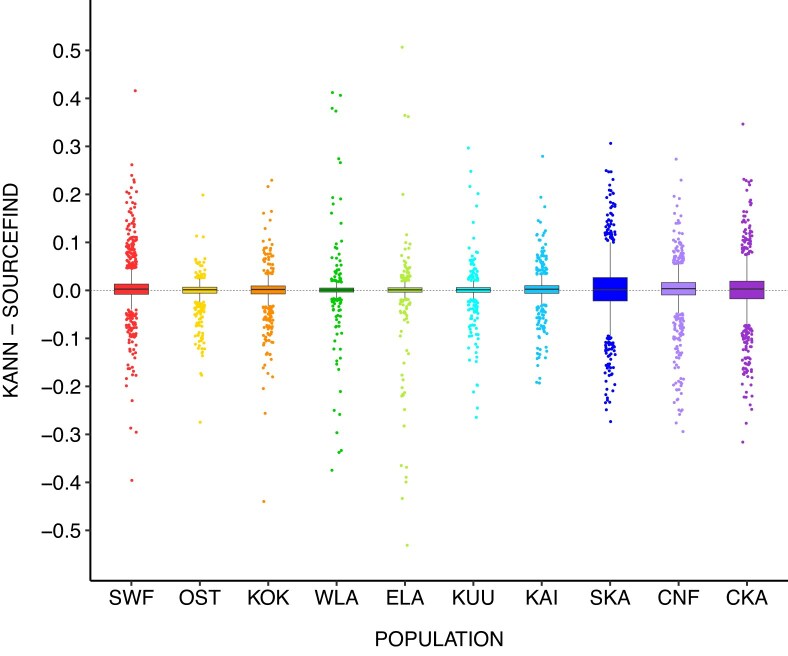
Marginal differences between KANN and SOURCEFIND in the ancestry components of the test set with respect to 10 source populations. The population abbreviations and colors are as in Fig. 1.

Population-specific marginal differences between KANN and SOURCEFIND with respect to the 10 source populations are depicted in Fig. 3. These are calculated from the test sample profiles using continuous ancestry information with the whole model-building dataset of 17 125 samples as the reference, and using the optimized parameters $(k = 25, p = 3)$. For all populations, the absolute values of the lower and upper quartiles remain $\le 0.04$. East Lapland (ELA), although having a small interquartile range, shows the largest positive (0.507) and negative (−0.531) differences. Supplementary Figs S13 and S14 show the marginal differences with respect to the 2 and 6 populations, respectively.

**Figure 4.**
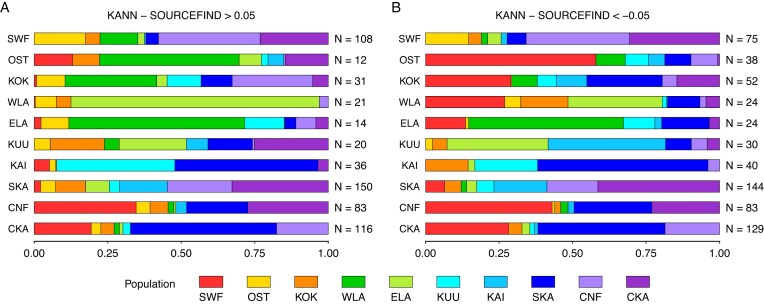
Distributions of the normalized absolute mean differences (*x*-axis) among the samples having a high (**A**) positive or a high (**B**) negative marginal difference in a particular target population. The *y*-axis shows the target population label (left-hand side), and the corresponding number of samples (right-hand side) reaching the threshold of 0.05 difference with respect to the target population. The population abbreviations and colors are given in Fig. 1.

Using the same estimated ancestry profiles, Fig. 4 visualizes the distributions of population-specific mean marginal differences among the samples having a high marginal difference in a particular target population (Supplementary Table S1). Supplementary Fig. S15 depicts the distributions with respect to six populations.

In general, population pairs that are geographical neighbors (Fig. 1) were most prone to getting mixed up between KANN and SOURCEFIND. Fig. 4 also indicates that the distributions in panels A and B are not entirely symmetrical. For example, among the individuals with a high positive difference in the West Lapland (WLA) component (row 4 in Fig. 4A), the vast majority (84.5%) of the ancestry was mixed up with its geographical neighbor, East Lapland (ELA), with virtually no mix-up (0.3%) with the geographically more distant Southwestern Finland (SWF). On the other hand, among the individuals with a high negative difference in the WLA component (row 4 in Fig. 4B), the ancestry has been mixed up substantially less with ELA (32.1%), while almost a comparable fraction (26.9%) of ancestry was estimated for the SWF component.

### Haplotype component results

Haplotype components obtained using PBWTpaint distinguish clear population structure in the Finnish data, as shown in Supplementary Figs S16–S18. Supplementary Fig. S19 shows the mean TVD observed across the parameter values during the optimization in the HCA model-building dataset.

The minimum mean TVD observed in the query sample set with different numbers of haplotype components is shown in Supplementary Fig. S7. The general trend across the different scenarios is analogous to PCs, where the TVD decreases rapidly after the inclusion of the second and third HC, and stagnates after approximately eight components. Regardless of the setting, using only the first PC yields better results than the first HC. However, after the inclusion of more components, HCs tend to give at least as low mean TVDs as the PCs, and slightly outperform them in some cases. For example, in the KANN-CONTINUOUSALL scenario, HCs result in a smaller minimum mean TVD than PCs after the third component for any number of source populations, although the difference is never more than 0.028 TVD units.

### 1000 Genomes Project results

During the parameter optimization process in the 1KGP dataset, KANN was used to estimate ancestry profiles for the 47 CLM query samples with respect to the five source populations that ADMIXTURE had previously inferred from the 1KGP data. Supplementary Fig. S20A shows the mean TVD observed between KANN (with 10 PCs) and ADMIXTURE in the query dataset with different parameter values. The minimum mean TVD across parameters as a function of the number of top PCs is shown in Supplementary Fig. S20B. After just the first two PCs, the minimum mean TVD decreases rapidly from around 0.22 and remains at approximately 0.03, with a slight increase after including more than 14 top PCs.

**Figure 5.**
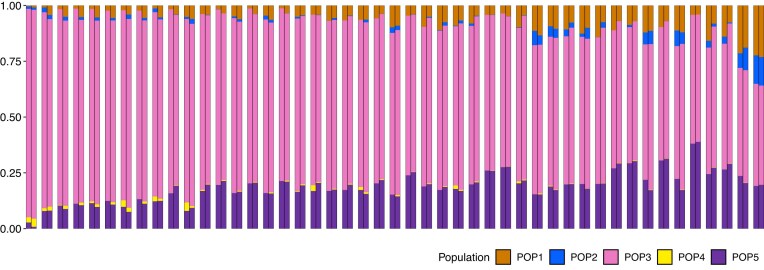
Ancestry profiles for the 47 CLM test samples with respect to the five populations inferred by ADMIXTURE. Each pair of bars represents one sample, where the left bar is the sample’s ADMIXTURE profile, and the right bar is the profile estimated using KANN $(k = 10, p = 5, 10 \text{ PCs})$.

Providing KANN with 10 PCs and the corresponding optimal parameter pair $(k = 10, p = 5)$, the mean TVD in the test set of 47 CLM samples was 0.0391. Fig. 5 shows these KANN profiles side by side with the original ADMIXTURE profiles. The scatter plots of population-specific test set ancestry estimates inferred by KANN and ADMIXTURE are depicted in Supplementary Fig. S21.

## Discussion

We developed KANN with the aim to offer a user-friendly and scalable tool that could produce similar results as computationally more complex haplotype-based ancestry estimation methods such as SOURCEFIND [5]. Our results indicate that KANN is able to estimate ancestry profiles that are well in line with SOURCEFIND. For example, with up to six source populations, all ancestry components from KANN and SOURCEFIND were highly correlated ($> 0.96$). Our analysis of the Finnish data demonstrated the performance of KANN in a single, relatively homogeneous European population. Additional benchmarking against ancestry profiles from the 1KGP ADMIXTURE analysis suggested that KANN is capable of estimating ancestry profiles highly consistent with ADMIXTURE in a dataset representing more diverse global populations (Fig. 5).

Naturally, the average difference between KANN and SOURCEFIND increases as we attempt to assign the Finnish ancestry into 10 fine-scale source populations. For many target populations, the difference between KANN and SOURCEFIND was nearly independent of which one gave a higher ancestry estimate for these target populations, as indicated by the similarity of panels A and B of Fig. 4. In these cases, the largest differences between KANN and SOURCEFIND tended to occur between source populations that are genetically close to each other as measured by the Fst values reported by Kerminen et al. in their Supplementary Table S2 [17]. Instead, for three geographically western populations, OST, KOK, and WLA, there was a clear asymmetry between panels A and B of Fig. 4. Compared to SOURCEFIND, KANN tended to replace some ancestry in these three populations by the SWF component (Fig. 4B) that represents the canonical Western Finland component, whereas for individuals for whom KANN’s estimate in these three populations was larger than SOURCEFIND’s estimate, the extra ancestry was mainly taken from one of the neighboring populations but not from SWF (Fig. 4A). Such asymmetry could reflect both complex relationships between the western source populations and properties of the PCA used in the KANN algorithm that may emphasize particular aspects of the population structure of western Finland.

In our Finnish dataset, no ground truth of ancestry fractions exists, and we evaluated the accuracy of the methods by comparing them to ancestry estimates from a well-established haplotype-based method, SOURCEFIND. We acknowledge that the improved similarity with respect to SOURCEFIND does not automatically imply greater accuracy relative to ancestry fractions defined using other methods. KANN was able to reproduce the SOURCEFIND’s estimates more accurately than an existing PCA-based ancestry estimation method, Rye [9], when using the same input data. Furthermore, we achieved a considerable improvement with KANN when we extended KANN to utilize continuous ancestry profiles of the reference individuals instead of assigning each reference individual to a single source population as required by Rye. Given that individuals from natural populations are often complex mixtures of different ancestries, we consider KANN’s ability to use the continuous ancestry profiles as an important advancement for many ancestry estimation applications. Since KANN is independent of the ancestry profile estimation method applied to the reference samples, the choice of the method can be made freely based on considerations of accuracy and computational feasibility [5, 6, 20, 21].

A practical question is how to choose a suitable number of principal components, and suitable values for the two parameters required by KANN: $k$ the number of nearest neighbors and $p$ the exponent of the inverse distance that is used to weight the $k$ nearest samples. If high-quality ancestry information can be estimated for a subset of the data, for example, by SOURCEFIND or some other haplotype-based method, the parameters can be optimized by using a cross-validation approach as we have demonstrated. If such information is not available, the parameters can alternatively be chosen based on some general trends we observed about the parameters’ effect on the estimation accuracy (Supplementary Fig. S5). The choice of $k$ and $p$ should be considered jointly, as their effects on estimation accuracy depend on each other. We acknowledge that the following observations are made in our dataset when running KANN with 10 PCs, and they might not generalize equally well in all other cases.

We observed that $p$ had a considerable effect only when $k$ was fairly large, say $> 5\%$ of the number of reference samples. There, increasing $p$ from 0 typically decreased the TVD to SOURCEFIND/ADMIXTURE. However, values of $p > 5$ did not anymore noticeably decrease the optimal TVD when we optimized $p$ and $k$ together. Hence, we consider a plausible range for $p$ to be between 0 and 5. In our Finnish data, fixing $p=3$ and optimizing over $k$ resulted, on average, in the smallest mean TVD over all optimization scenarios and populations when using 10 PCs (Supplementary Fig. S5). In the 1KGP dataset, $p=5$ resulted in the smallest mean TVD (Supplementary Fig. S20A) but the TVD achieved with $p=3$ had only a tiny (0.0006) difference from the optimum. Therefore, we propose $p=3$ as a default value for datasets where no parameter optimization is possible.

The optimal value of $k$ is also influenced by the size of the reference sample set $n_R$ and the genetic diversity among the data. In our Finnish examples, when $n_R < 1500$ (KANN-DISCRETE and KANN-CONTINUOUS), large values of $k$ were preferred as $k = 1000$ was the smallest optimal value observed. On the other hand, with the largest reference sample set with $n_R > 15 \, 000$ (KANN-CONTINUOUSALL), much smaller values $(k = 25, k = 50)$ were optimal. In the 1KGP analysis, where $n_R = 2410$ but the genetic diversity is considerably larger than in the Finnish data, we observed $k=10$ to be optimal. However, even larger values of $k$ with varying levels of distance weighting produced near optimal results, suggesting that a large reference sample set does not necessarily mean that smaller values of $k$ should always be preferred. For example, in the Finnish data, the difference between the optimal mean TVD and the one obtained using, say, $k = 10 \, 000$ and $p = 5$ in the KANN-CONTINUOUSALL scenario is at most 0.036, regardless of the number of populations. If no distance weighting is used ($p = 0$), then we recommend avoiding values of $k$ that are close to the full reference dataset size, because otherwise the ancestry estimates of all individuals would become very similar as they would be (unweighted) averages over largely overlapping sets of the reference individuals.

Here, we ran our main analyses with KANN using the first 10 PCs, and confirmed it to be a suitable number in our examples. Using only the first PC yielded the worst results, and in most cases, inclusion of more components decreased the TVD up to a point, depending on the optimization scenario and number of populations (Supplementary Fig. S7). In the Finnish data, with only two source populations, TVD did not show a considerable change after the first five PCs. With 10 source populations, we saw a steady decrease in TVD that stagnates after approximately the first eight PCs. Beyond the first 10 PCs, no substantial further change in TVD was observed. In the 1KGP dataset with five source populations, including only the first three principal components, yielded optimal results (Supplementary Fig. S20B), and no considerable change was seen in the TVD after inclusion of further components. Unless dataset-specific optimization has been carried out for the number of PCs, we suggest 10 PCs as a default choice for running KANN, as that number gave results that were always very near the optimum observed.

As ancestry estimation with KANN is based on PCs, it is important to carefully conduct the data preprocessing steps to achieve a high-quality PCA [11, 12, 22]. The effect of LD pruning and choice of variants on the PCA will vary in different settings, and therefore, visual inspection of the resulting PC scores with respect to known ancestry-related labels is always important. In our work, we considered common variants with MAF $\ge$ 5%. Alternative variant selection strategies (e.g. inclusion of low-frequency or rare variants) might emphasize different features of population structure, especially when considering recent admixture [23]. Furthermore, we left F-outliers and relatives starting from the third degree out from PCA to avoid possible family structure effects, but later projected them on the PCA space to increase the sample size. The projected samples are known to be shrunk towards the mean point of the PCs, and in some cases, this can affect the results [22]. This issue should be borne in mind in future applications of KANN.

Ancestry estimation with SOURCEFIND and similar software works with the output from chromosome painting software, including ChromoPainter [18] and its sparse approximation, SparsePainter [10]. Running ChromoPainter requires the painting of each query haplotype locus against every reference individual, corresponding to between 1026 (refset 6) and 18 125 (full data) comparisons per query individual in our dataset. SparsePainter approximates this process by limiting comparisons to a smaller number of best-matching reference haplotypes, but still relies on genome-wide haplotype matching. PC-like components enable the fast comparison between query and reference sample coordinates over only a handful of components.

We found that running KANN using PBWTpaint-derived HCs based on dense haplotype data showed high concordance with the estimates obtained with PCs extracted from a much smaller set of nearly LD-independent variants. In many cases, HCs slightly improved the estimation accuracy, as measured by mean TVD in the query dataset. However, an advantage of running KANN with PCs is to enable the fast projection (and the subsequent ancestry estimation) of new individuals to the existing PC-space using genotype loadings, without affecting the PC-coordinates of any other sample. To our knowledge, a similar projection for a new individual is not available for HCs extracted using PBWTpaint without recomputing the haplotype sharing with respect to all reference samples. Nevertheless, we recognize haplotype components as a plausible alternative to principal components in specific applications of KANN, when dense haplotype data and computational resources are available.

Obvious future application areas of KANN include population genetic studies and the individuals’ own interest in their personal genetic ancestry through direct-to-consumer services. Additionally, we expect that detailed information on individual-level genetic ancestry on biobank-scale datasets will enable new epidemiological studies to separate the effect of genetic ancestry from environmental factors. Finally, since the genetic ancestry profile does affect the performance of polygenic scores [2, 3], KANN can help us to choose the most suitable genetic predictions for each individual according to the individual’s detailed ancestry profile.

## Supplementary Material

gkag209_Supplemental_Files

## Data Availability

KANN is available as a software package in R-language at https://github.com/riikonenj/KANN and https://doi.org/10.5281/zenodo.18670452. The genotype data from Finnish cohorts used in this study are available from THL biobank via procedures outlined in the Finnish Biobank Act, and access can be obtained for biomedical research by contacting admin.biobank@thl.fi. The 1KGP genotypes and corresponding ADMIXTURE ancestry fractions are available at https://ftp-trace.ncbi.nih.gov/1000genomes/ftp/release/20130502/supporting/admixture_files/.

## References

[1] Jermy B, Läll K, Wolford BN et al. A unified framework for estimating country-specific cumulative incidence for 18 diseases stratified by polygenic risk. Nat Commun. 2024;15:5007. 10.1038/s41467-024-48938-2. 38866767 PMC11169548

[2] Martin AR, Kanai M, Kamatani Y et al. Clinical use of current polygenic risk scores may exacerbate health disparities. Nat Genet. 2019;51:584–91. 10.1038/s41588-019-0379-x. 30926966 PMC6563838

[3] Ndong Sima CAA, Step K, Swart Y et al. Methodologies underpinning polygenic risk scores estimation: a comprehensive overview. Hum Genet. 2024;143:1–16. 39425790 10.1007/s00439-024-02710-0 PMC11522080

[4] Jiang S, Liberti L, Lebo D. Direct-to-consumer genetic testing: a comprehensive review. Ther Innov Regul Sci. 2023;57:1190–8. 10.1007/s43441-023-00567-5. 37589855

[5] Chacón-Duque JC, Adhikari K, Fuentes-Guajardo M et al. Latin Americans show wide-spread Converso ancestry and imprint of local Native ancestry on physical appearance. Nat Commun. 2018;9:5388. 10.1038/s41467-018-07748-z. 30568240 PMC6300600

[6] Maples BK, Gravel S, Kenny EE et al. RFMix: a discriminative modeling approach for rapid and robust local-ancestry inference. Am J Hum Genet. 2013;93:278–88. 10.1016/j.ajhg.2013.06.020. 23910464 PMC3738819

[7] Bansal V, Libiger O. Fast individual ancestry inference from DNA sequence data leveraging allele frequencies for multiple populations. BMC Bioinf. 2015;16:1–11. 10.1186/s12859-014-0418-7. PMC4301802 25592880

[8] Alexander DH, Novembre J, Lange K. Fast model-based estimation of ancestry in unrelated individuals. Genome Res. 2009;19:1655–64. 10.1101/gr.094052.109. 19648217 PMC2752134

[9] Conley AB, Rishishwar L, Ahmad M et al. Rye: genetic ancestry inference at biobank scale. Nucleic Acids Res. 2023;51:e44. 10.1093/nar/gkad149. 36928108 PMC10164567

[10] Yang Y, Durbin R, Iversen AK et al. Sparse haplotype-based fine-scale local ancestry inference at scale reveals recent selection on immune responses. Nat Commun. 2025;16:2742. 10.1038/s41467-025-57601-3. 40113767 PMC11926123

[11] Price AL, Zaitlen NA, Reich D et al. New approaches to population stratification in genome-wide association studies. Nat Rev Genet. 2010;11:459–63. 10.1038/nrg2813. 20548291 PMC2975875

[12] Abegaz F, Chaichoompu K, Génin E et al. Principals about principal components in statistical genetics. Brief Bioinform. 2019;20:2200–16. 10.1093/bib/bby081. 30219892

[13] Chang CC, Chow CC, Tellier LC et al. Second-generation PLINK: rising to the challenge of larger and richer datasets. GigaScience. 2015;4:s13742–015-0047-8. 10.1186/s13742-015-0047-8. PMC4342193 25722852

[14] Ma S, Shi G. On rare variants in principal component analysis of population stratification. BMC genetics. 2020;21:34. 10.1186/s12863-020-0833-x. 32183706 PMC7077175

[15] Price AL, Weale ME, Patterson N et al. Long-range LD can confound genome scans in admixed populations. Am J Hum Genet. 2008;83:132–5. 10.1016/j.ajhg.2008.06.005. 18606306 PMC2443852

[16] Manichaikul A, Mychaleckyj JC, Rich SS et al. Robust relationship inference in genome-wide association studies. Bioinformatics. 2010;26:2867–73. 10.1093/bioinformatics/btq559. 20926424 PMC3025716

[17] Kerminen S, Cerioli N, Pacauskas D et al. Changes in the fine-scale genetic structure of Finland through the 20th century. PLoS Genet. 2021;17:e1009347. 10.1371/journal.pgen.1009347. 33661898 PMC7932171

[18] Lawson DJ, Hellenthal G, Myers S et al. Inference of population structure using dense haplotype data. PLoS Genet. 2012;8:e1002453. 10.1371/journal.pgen.1002453. 22291602 PMC3266881

[19] Consortium GP . A global reference for human genetic variation. Nature. 2015;526:68. 10.1038/nature15393. 26432245 PMC4750478

[20] Salter-Townshend M, Myers S. Fine-scale inference of ancestry segments without prior knowledge of admixing groups. Genetics. 2019;212:869–89. 10.1534/genetics.119.302139. 31123038 PMC6614886

[21] Browning SR, Waples RK, Browning BL. Fast, accurate local ancestry inference with FLARE. Am J Hum Genet. 2023;110:326–5. 10.1016/j.ajhg.2022.12.010. 36610402 PMC9943733

[22] Privé F, Luu K, Blum MG et al. Efficient toolkit implementing best practices for principal component analysis of population genetic data. Bioinformatics. 2020;36:4449–57. 10.1093/bioinformatics/btaa520. 32415959 PMC7750941

[23] Zaidi AA, Mathieson I. Demographic history mediates the effect of stratification on polygenic scores. Elife. 2020;9:e61548. 10.7554/eLife.61548. 33200985 PMC7758063

