# Supplementary material for: KANN: estimation of genetic ancestry profiles by nearest neighbor regression
Source: Nucleic Acids Res. 2026 Mar 12;54(5):gkag209. doi: 10.1093/nar/gkag209 (PMC12980074; doi:10.1093/nar/gkag209)
Supplement: gkag209_Supplemental_Files [file gkag209_supplemental_files.zip › KANN_Supplementary_material_revision_2.pdf]

Supplementary material for

KANN: estimation of genetic ancestry profiles by nearest neighbor regression

Juha Riikonen[1,*], Sini Kerminen[1], Aki Havulinna[1,2], and Matti Pirinen[1,3,4,*]

[1]*Institute for Molecular Medicine Finland, Helsinki Institute of Life Science, University of Helsinki, Helsinki, Finland*

[2]*Department of Computing, University of Turku, Turku, Finland*

[3]*Department of Public Health, University of Helsinki, Helsinki, Finland*

[4]*Department of Mathematics and Statistics, University of Helsinki, Helsinki, Finland*

List of Figures

S1 Supplementary Figure S1 1
S2 Supplementary Figure S2 2
S3 Supplementary Figure S3 3
S4 Supplementary Figure S4 4
S5 Supplementary Figure S5 5
S6 Supplementary Figure S6 6
S7 Supplementary Figure S7 7
S8 Supplementary Figure S8 8
S9 Supplementary Figure S9 9
S10 Supplementary Figure S10 10
S11 Supplementary Figure S11 11
S12 Supplementary Figure S12 12
S13 Supplementary Figure S13 13
S14 Supplementary Figure S14 14
S15 Supplementary Figure S15 15
S16 Supplementary Figure S16 16
S17 Supplementary Figure S17 17
S18 Supplementary Figure S18 18
S19 Supplementary Figure S19 19
S20 Supplementary Figure S20 20
S21 Supplementary Figure S21 21

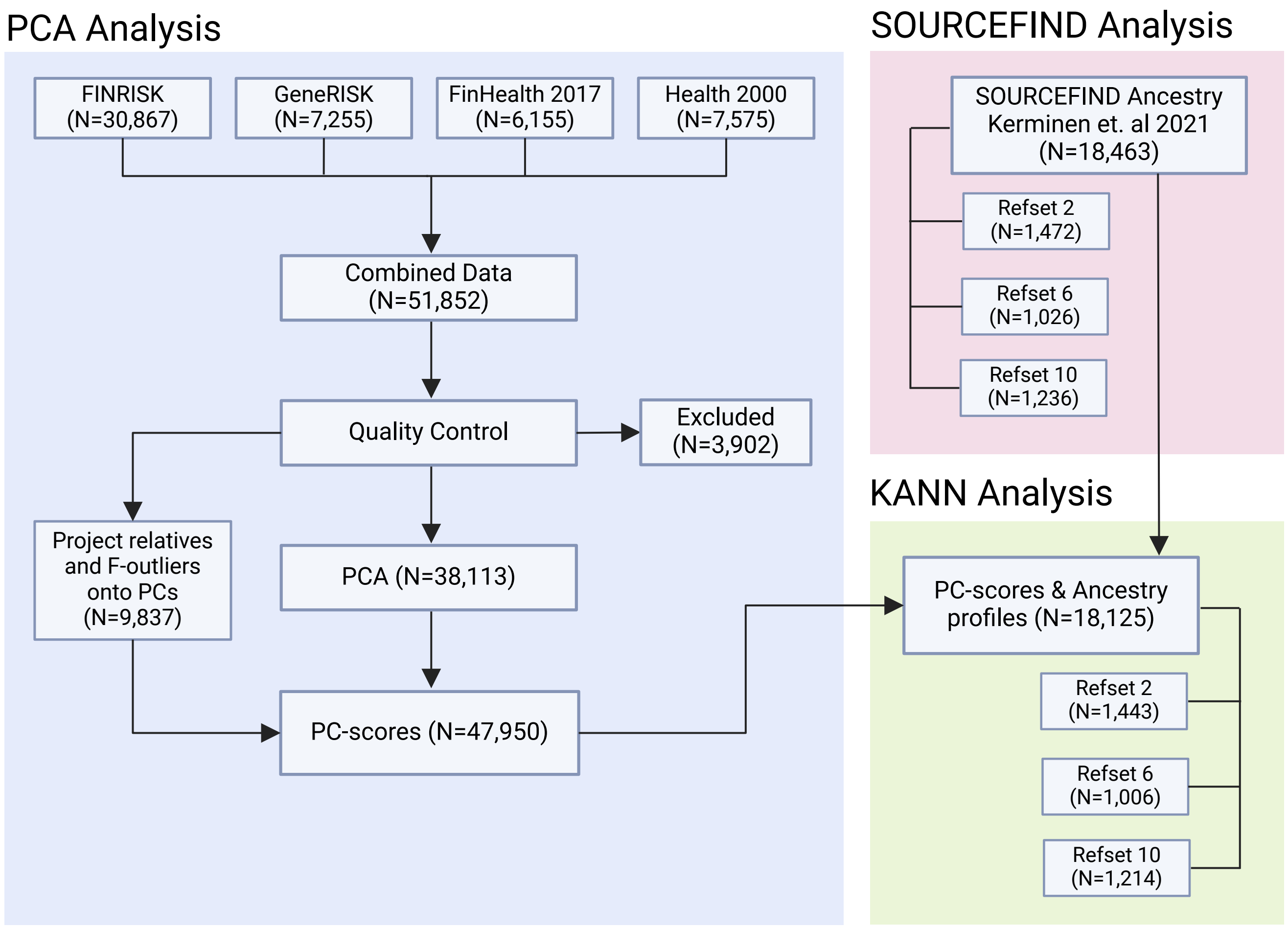

Supplementary Figure S1: Workflow of the study. Part "SOUCREFIND Analysis" was done by Kerminen et. al (2021). Figure created in BioRender. Riikonen, J. (2025) https://BioRender.com/k67z392

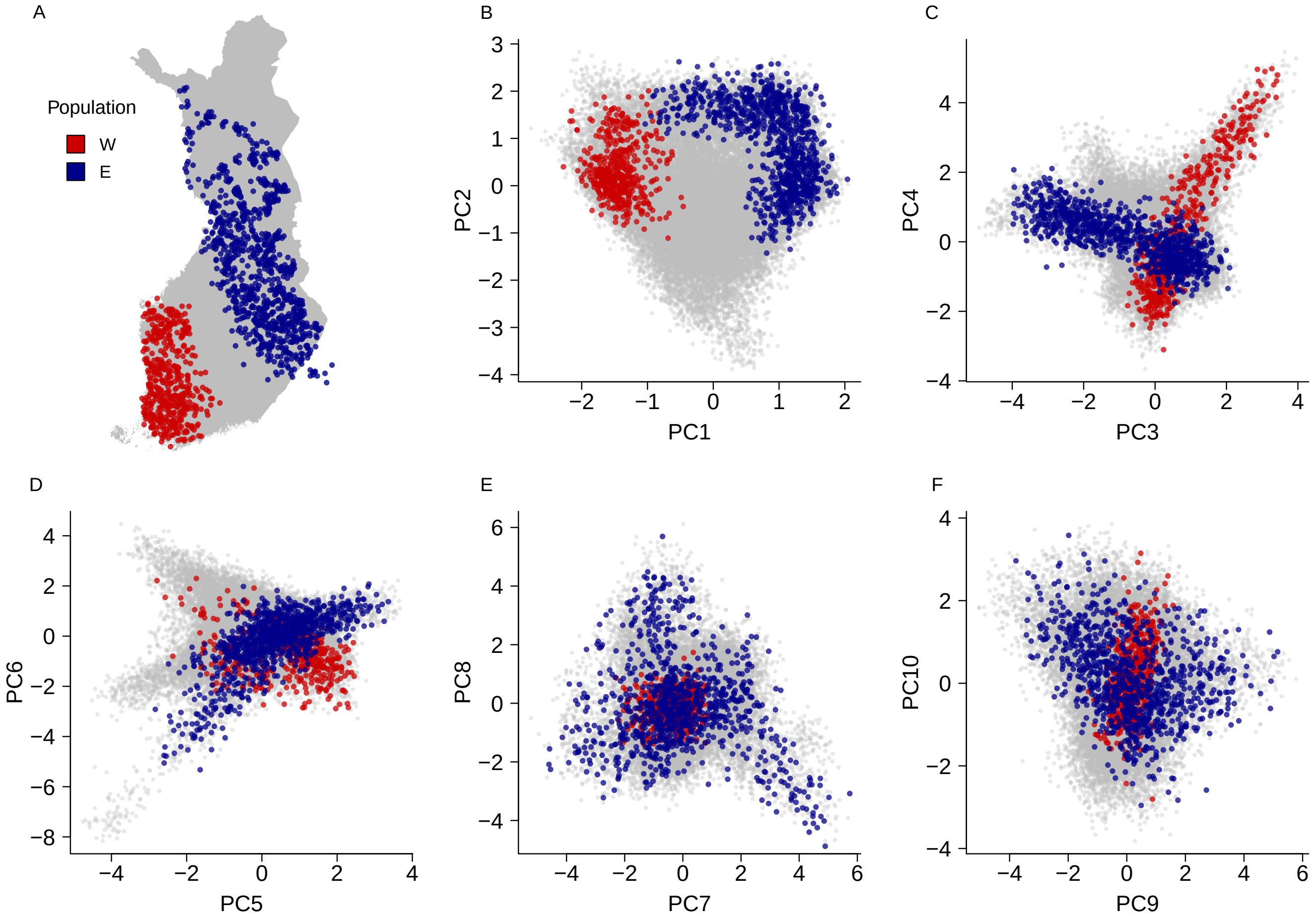

Supplementary Figure S2: A) Map of Finland with the geographical locations of the reference samples (n = 1,443) allocated to 2 Finnish source populations. The points depict the mean coordinates of the parents' municipalities of birth. B-F) The same individuals highlighted on the first ten principal components. The samples not included in the reference groups are depicted in grey colour. W: West, E: East.

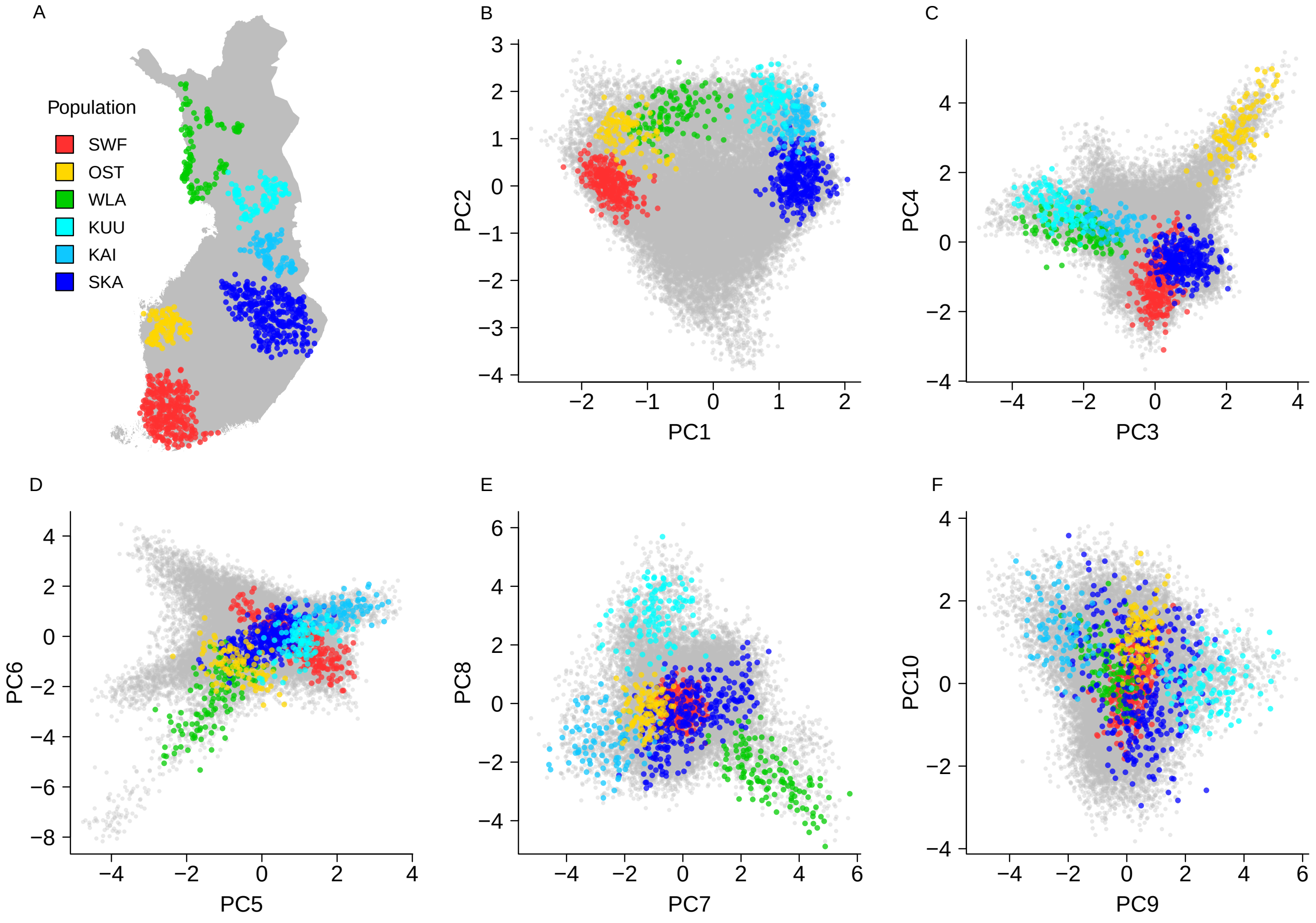

Supplementary Figure S3: A) Map of Finland with the geographical locations of the reference samples (n = 1,006) allocated to 6 Finnish source populations. The points depict the mean coordinates of the parents' municipalities of birth. B-F) The same individuals highlighted on the first ten principal components. The samples not included in the reference groups are depicted in grey colour. SWF: Southwestern Finland, OST: Ostrobothnia, WLA: West Lapland, KUU: Kuusamo, KAI: Kainuu, SKA: Savo-Karelia.

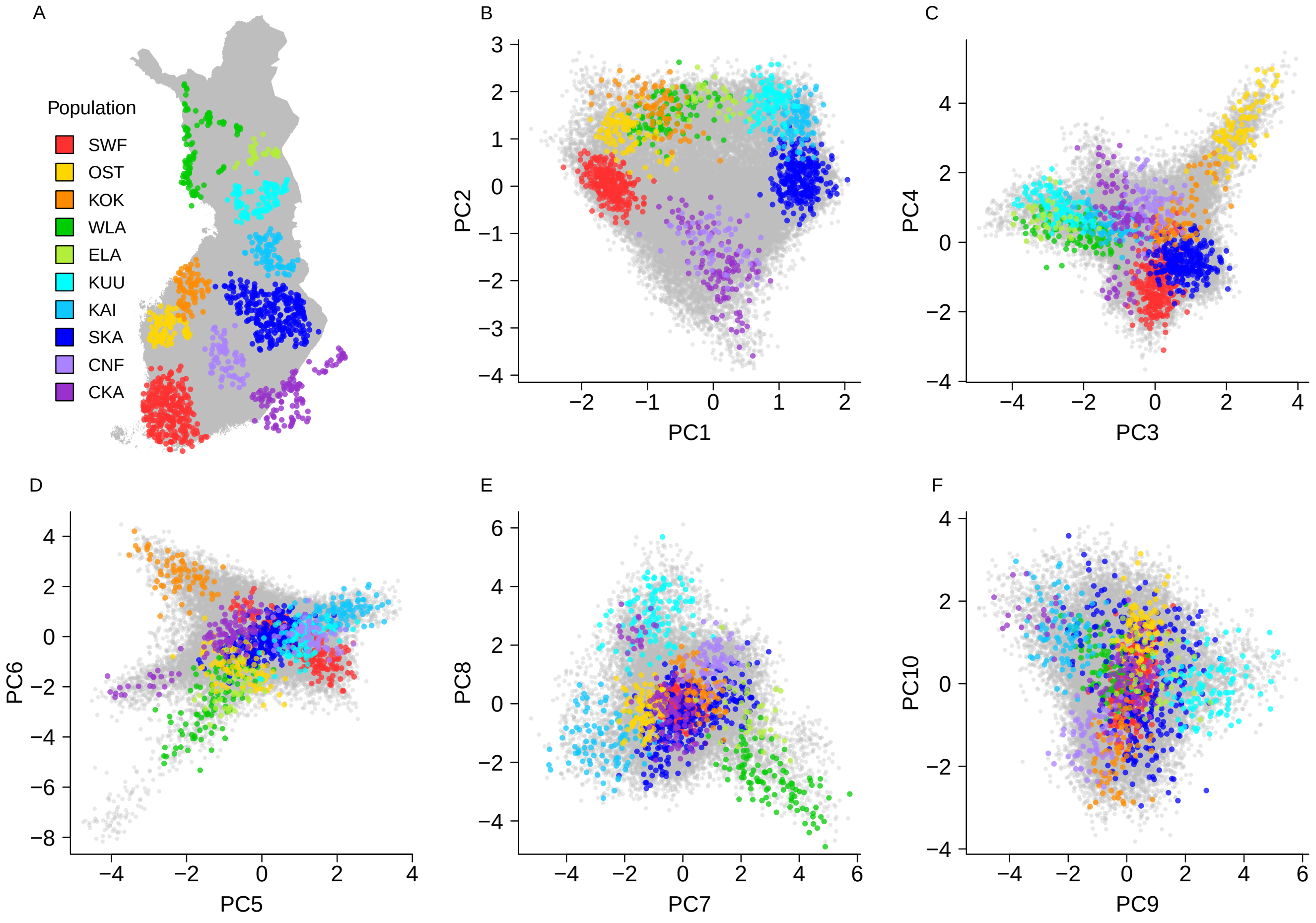

Supplementary Figure S4: A) Map of Finland with the geographical locations of the reference samples (n = 1,214) allocated to 10 Finnish source populations. The points depict the mean coordinates of the parents' municipalities of birth. B-F) The same individuals highlighted on the first ten principal components. The samples not included in the reference groups are depicted in grey colour. SWF: Southwestern Finland, OST: Ostrobothnia, KOK: Kokkola, WLA: West Lapland, ELA: East Lapland, KUU: Kuusamo, KAI: Kainuu, SKA: Savo-Karelia, CNF: Central Finland, CKA: Ceded Karelia.

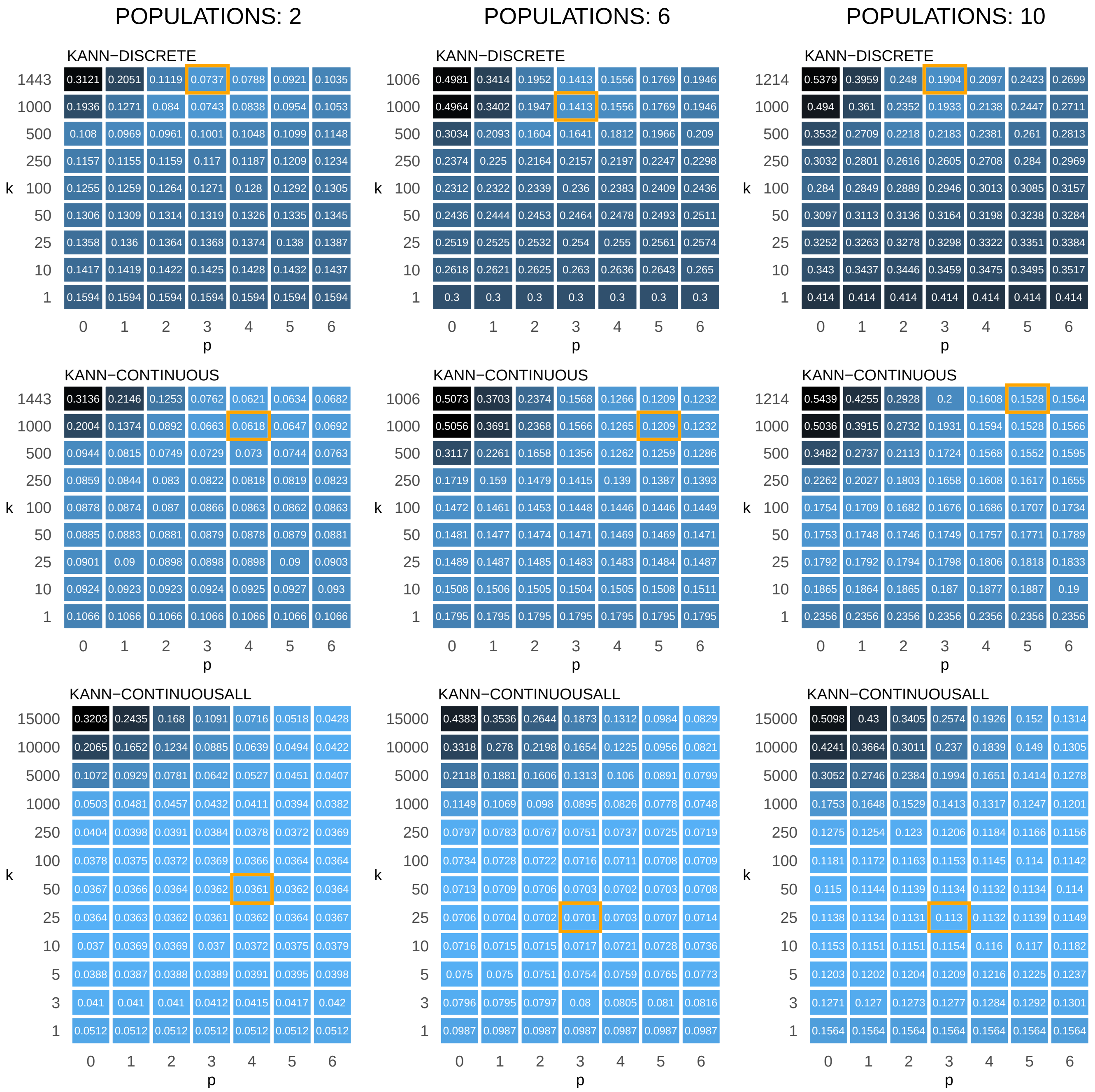

Supplementary Figure S5: Mean TVD of the query sample set profiles estimated using KANN with the first 10 principal components and all parameter configurations considered. For the parameter p, values are shown from 0 to 6, since we did not observe any optimal parameter pairs with larger values of p during the optimization process when using the first 10 PCs. The TVD is shown for three optimization scenarios (DISCRETE, CONTINUOUS, CONTINUOUSALL) and with different numbers of source populations (2, 6, 10). The values are rounded up to four decimals and the minimum TVD is highlighted.

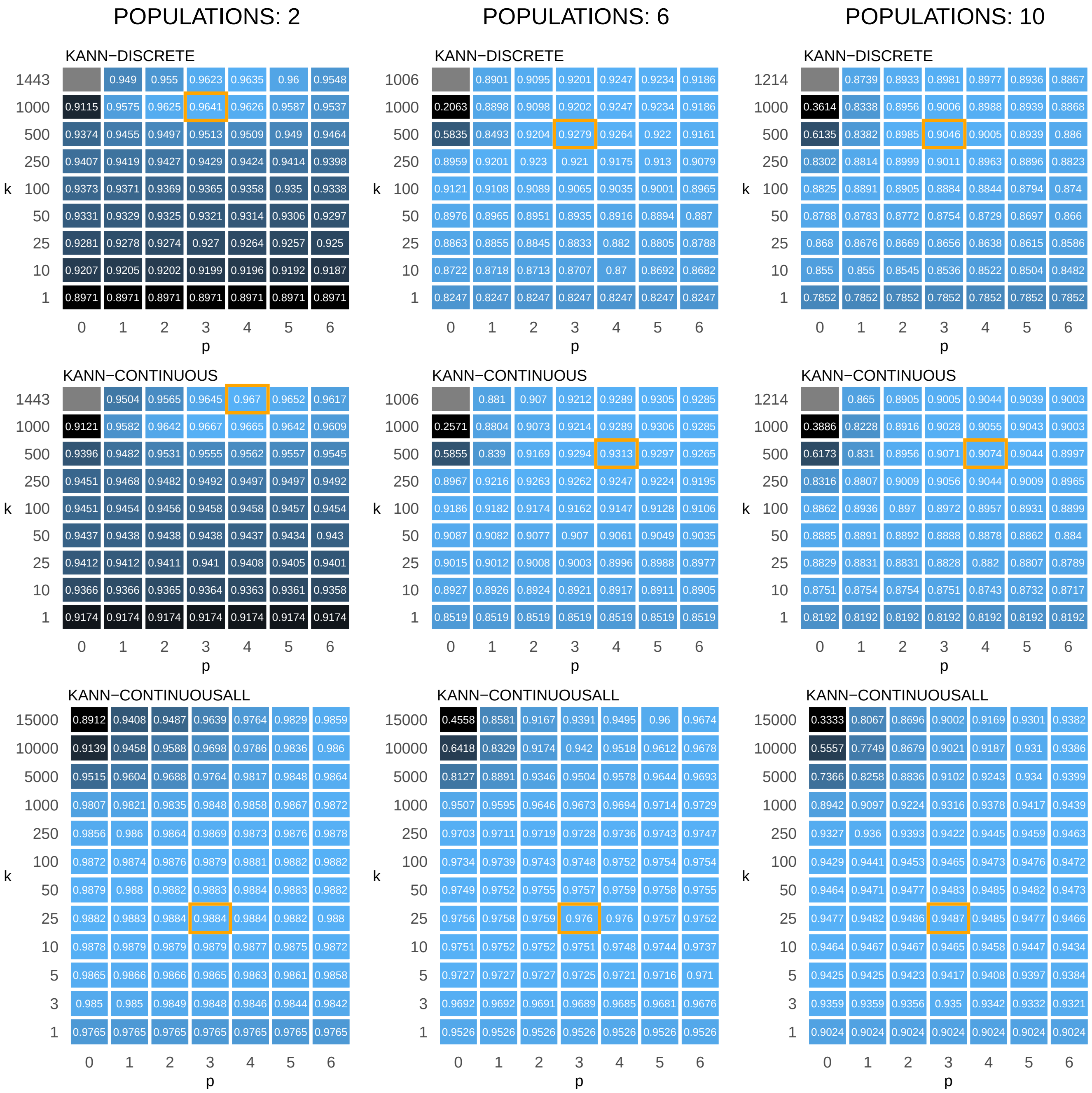

Supplementary Figure S6: Mean of the Pearson correlation coefficients over all ancestry components estimated for the query sample set profiles estimated using KANN with the first 10 principal components and all parameter configurations considered. For the parameter p, values are shown from 0 to 6, since we did not observe any optimal parameter pairs with larger values of p during the optimization process when using the first 10 PCs. The mean correlations are shown for three optimization scenarios (DISCRETE, CONTINUOUS, CONTINUOUSALL) and with different numbers of source populations (2, 6, 10). The values are rounded up to four decimals and the maximum correlation is highlighted.

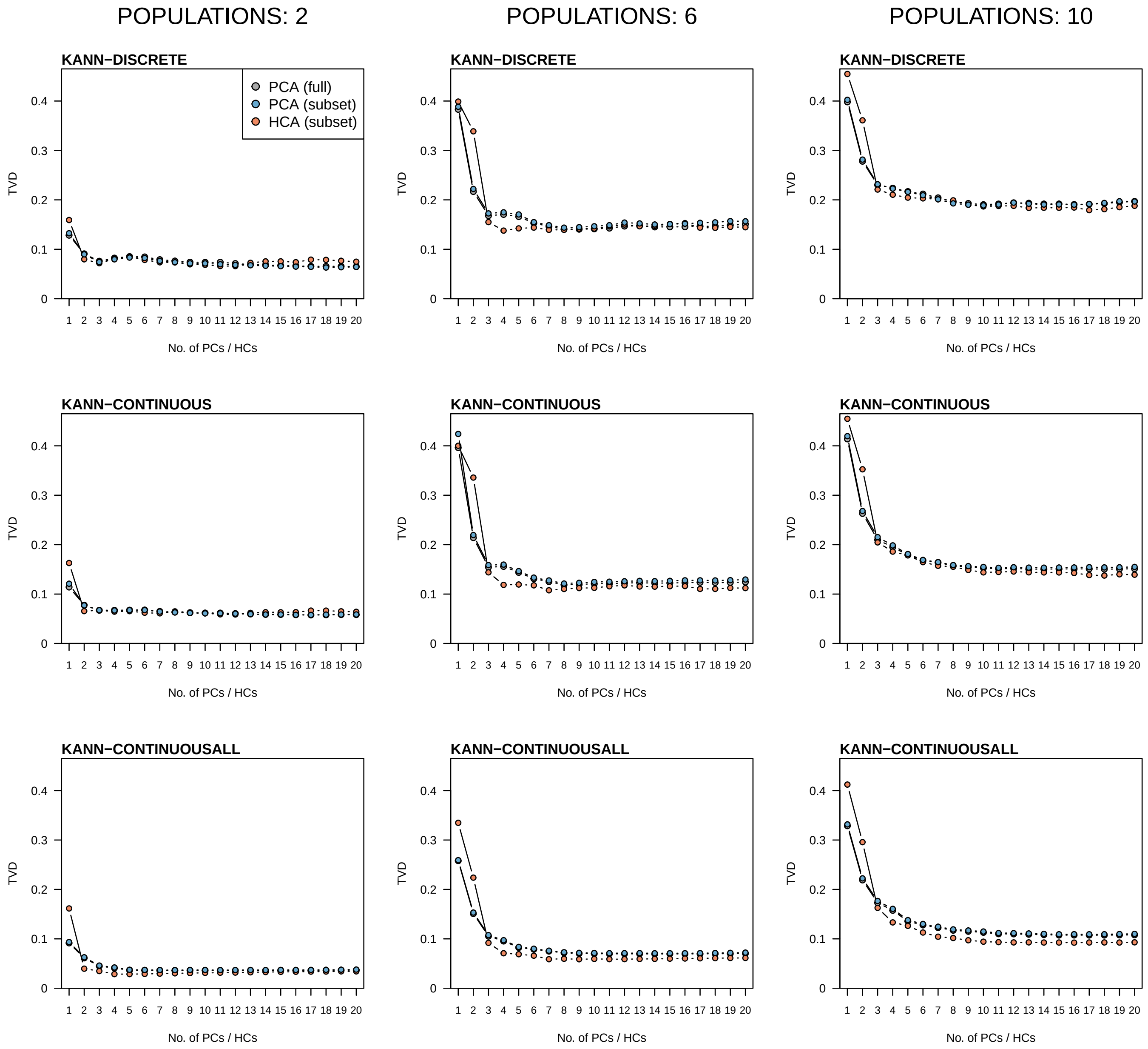

Supplementary Figure S7: The minimum mean TVD (y-axis) obtained in the query sample set across the parameter optimization process repeated with different number of components (x-axis). For PCs, the minimum mean TVD is shown from the optimization process using the full PCA model building data set as reference with the corresponding optimal parameters (gray). For HCs, the value is shown from the HC optimization process and the corresponding optimal parameters (red). Since the data set with HCs was smaller than data set with PCs, the figure includes also the results for PCs using the same reference samples as with HCs, with parameters from the PCA full data optimization (blue).

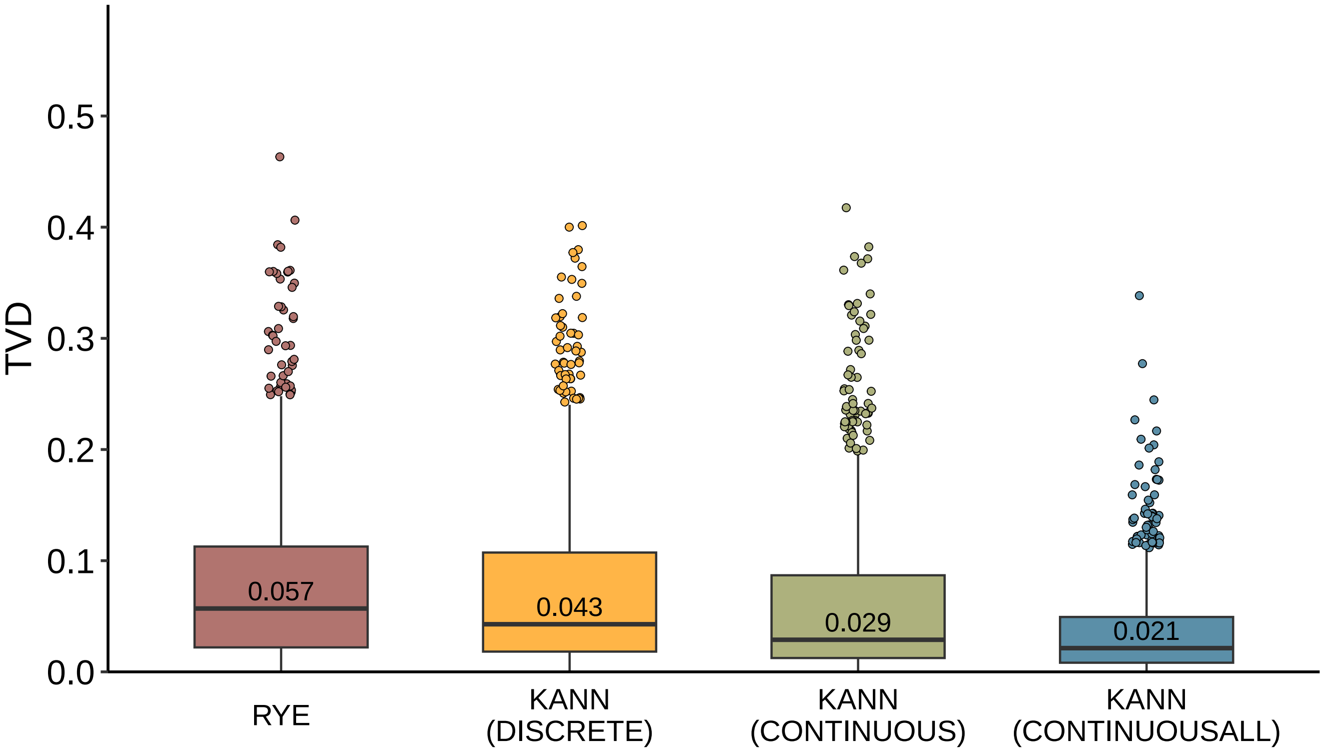

Supplementary Figure S8: Box plots showing the TVD distribution of the test sample profiles with respect to 2 populations. Profiles are estimated using Rye, and the three versions of KANN. The profiles in each version of KANN have been estimated using the optimal parameter pair. The median TVD is shown on each box plot.

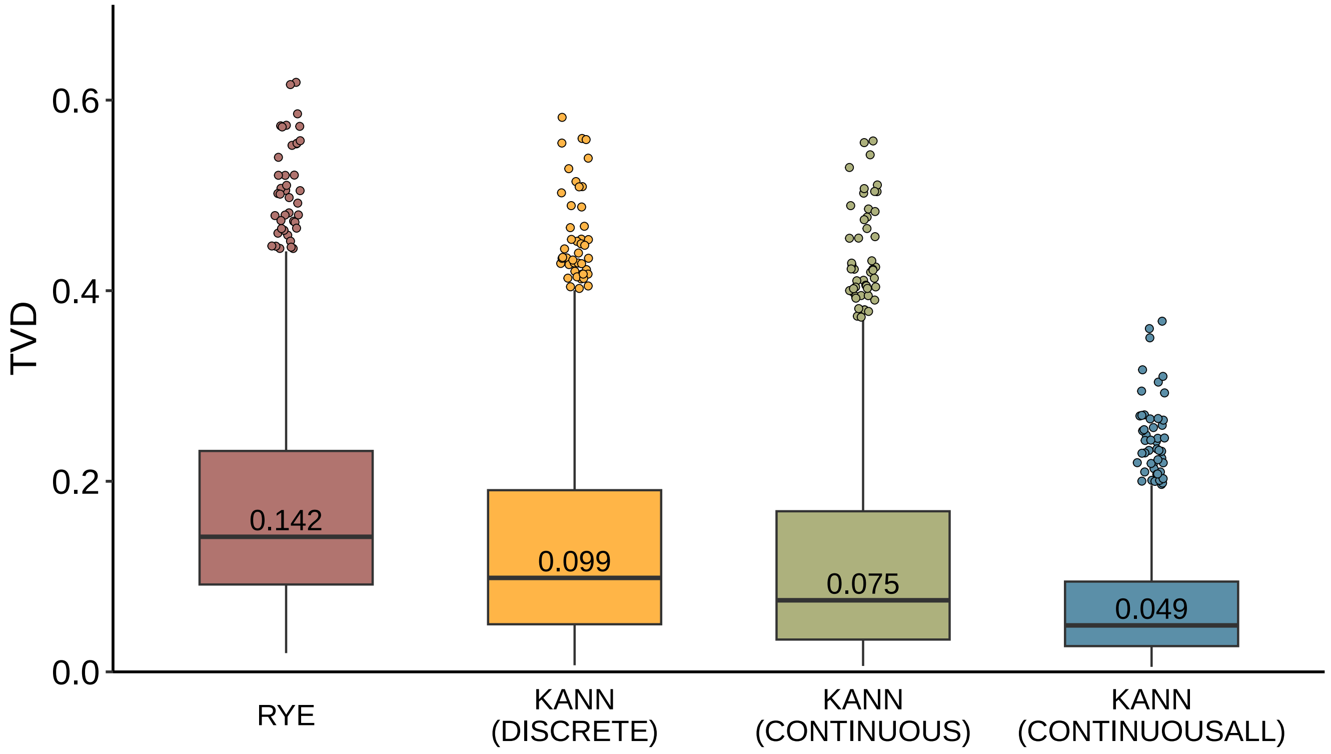

Supplementary Figure S9: Box plots showing the TVD distribution of the test sample profiles with respect to 6 populations. Profiles are estimated using Rye, and the three versions of KANN. The profiles in each version of KANN have been estimated using the optimal parameter pair. The median TVD is shown on each box plot.

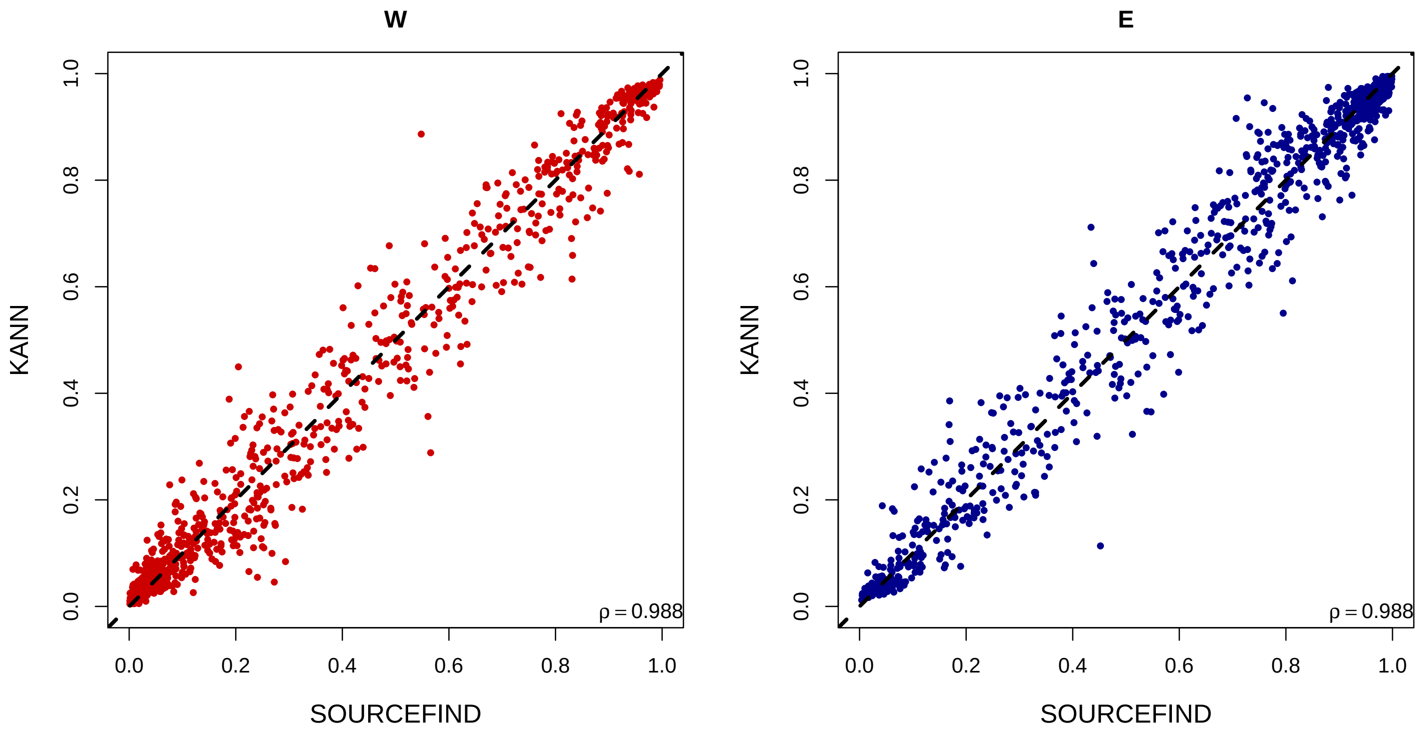

Supplementary Figure S10: Pairwise scatterplots of the test set samples' ancestry components estimated using KANN and SOURCEFIND for 2 source populations. Diagonal is shown as a dashed line. Each panel reports the Pearson correlation coefficient (ρ) between the estimates of the two methods. The population abbreviations and colors are given in Supplementary Fig. S2.

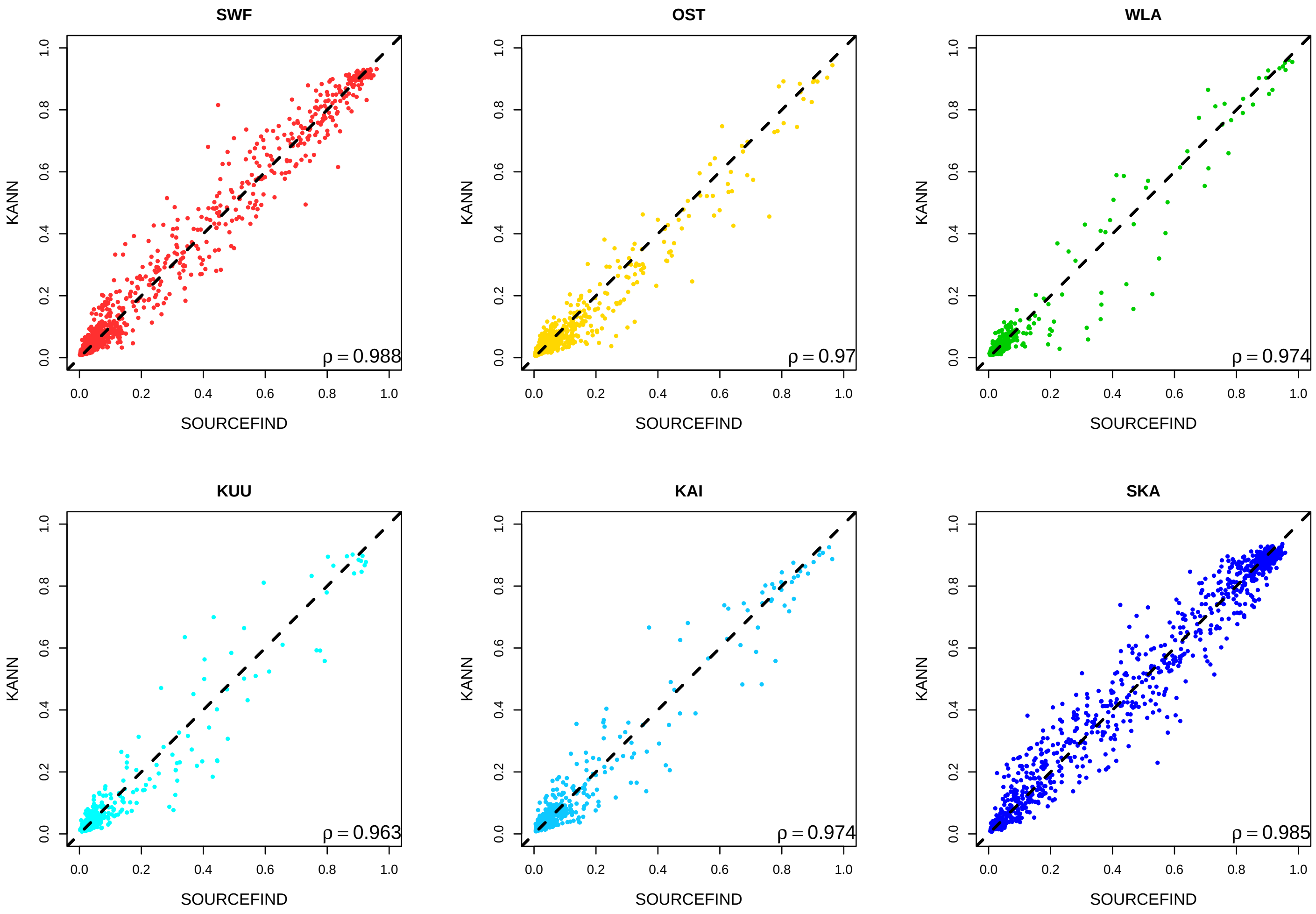

Supplementary Figure S11: Pairwise scatterplots of the test set samples' ancestry components estimated using KANN and SOURCEFIND for 6 source populations. Diagonal is shown as a dashed line. Each panel reports the Pearson correlation coefficient (ρ) between the estimates of the two methods. The population abbreviations and colors are given in Supplementary Fig. S3.

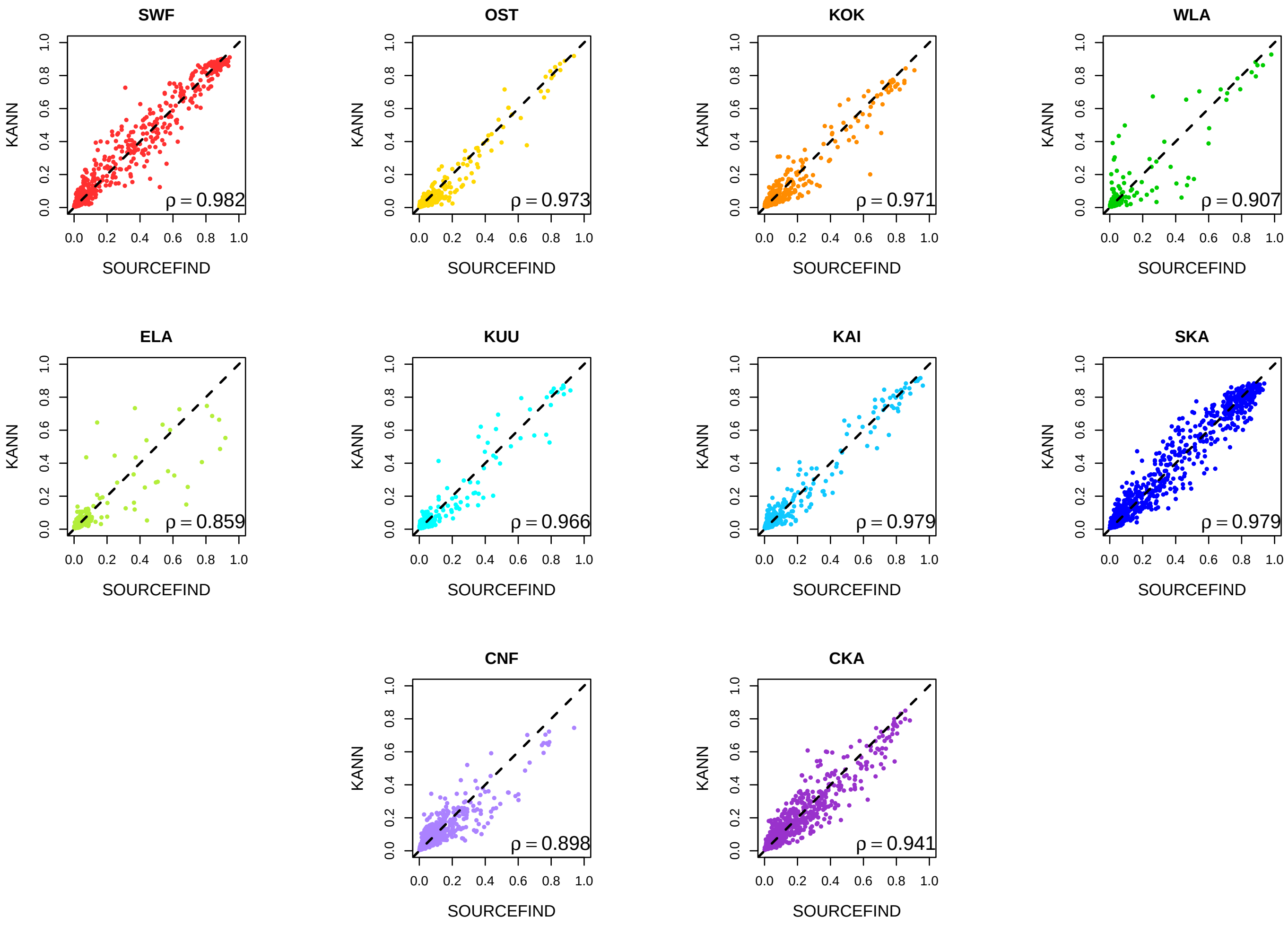

Supplementary Figure S12: Pairwise scatterplots of the test set samples' ancestry components estimated using KANN and SOURCEFIND for 10 source populations. Diagonal is shown as a dashed line. Each panel reports the Pearson correlation coefficient (ρ) between the estimates of the two methods. The population abbreviations and colors are given in Supplementary Fig. S4.

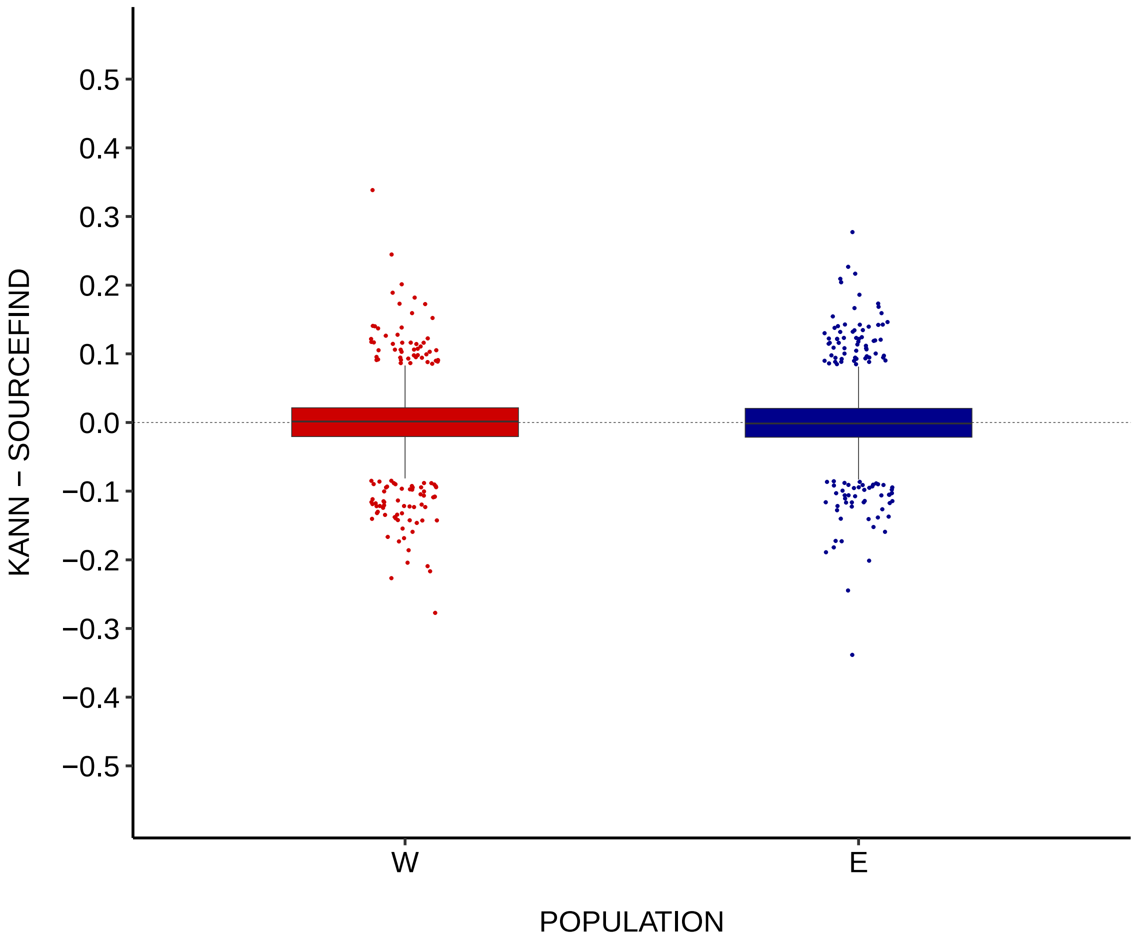

Supplementary Figure S13: Marginal differences between KANN and SOURCEFIND in the ancestry components of the test set with respect to 2 source populations. KANN profiles are estimated using 17,125 reference samples with continuous ancestry information, and the optimal parameter pair (k = 50, p = 4). The population abbreviations and colors are as in Supplementary Fig. S2.

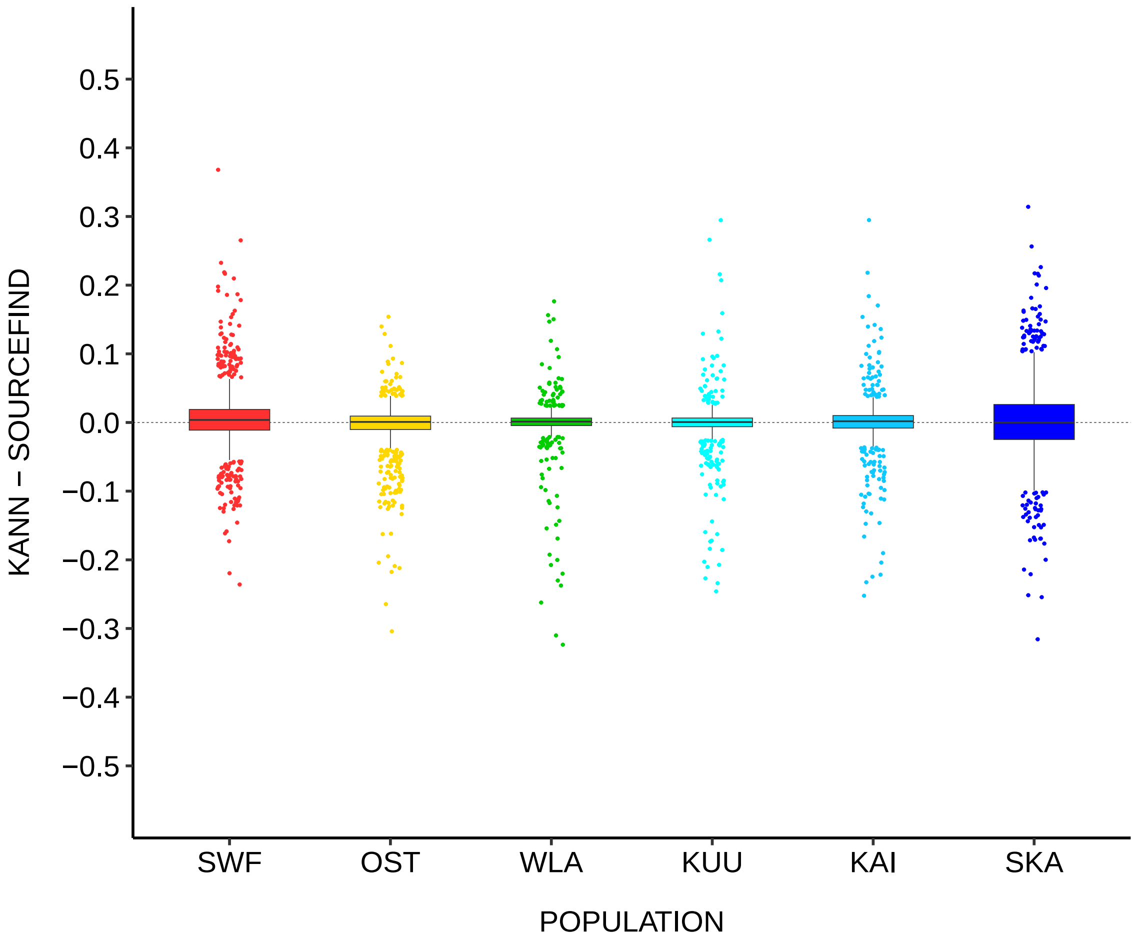

Supplementary Figure S14: Marginal differences between KANN and SOURCEFIND in the ancestry components of the test set with respect to 6 source populations. KANN profiles are estimated using 17,125 reference samples with continuous ancestry information, and the optimal parameter pair (k = 25, p = 3). The population abbreviations and colors are as in Supplementary Fig. S3.

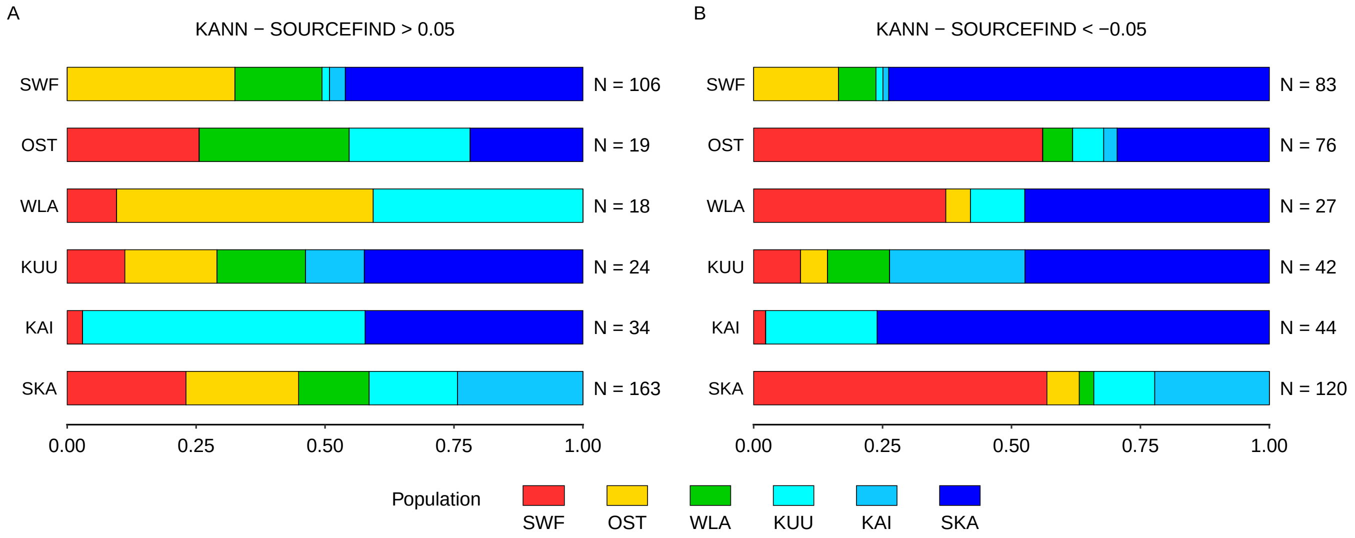

Supplementary Figure S15: Distributions of the normalized absolute mean differences (x-axis) among the samples having a high A) positive or a high B) negative marginal difference in a particular target population. The y-axis shows the target population label (left-hand side), and the corresponding number of samples (right-hand side) reaching the threshold of 0.05 difference with respect to the target population. KANN profiles are estimated using 17,125 reference samples with continuous ancestry information, and the optimal parameter pair (k = 25, p = 3). The population abbreviations and colors are given in Supplementary Fig. S3.

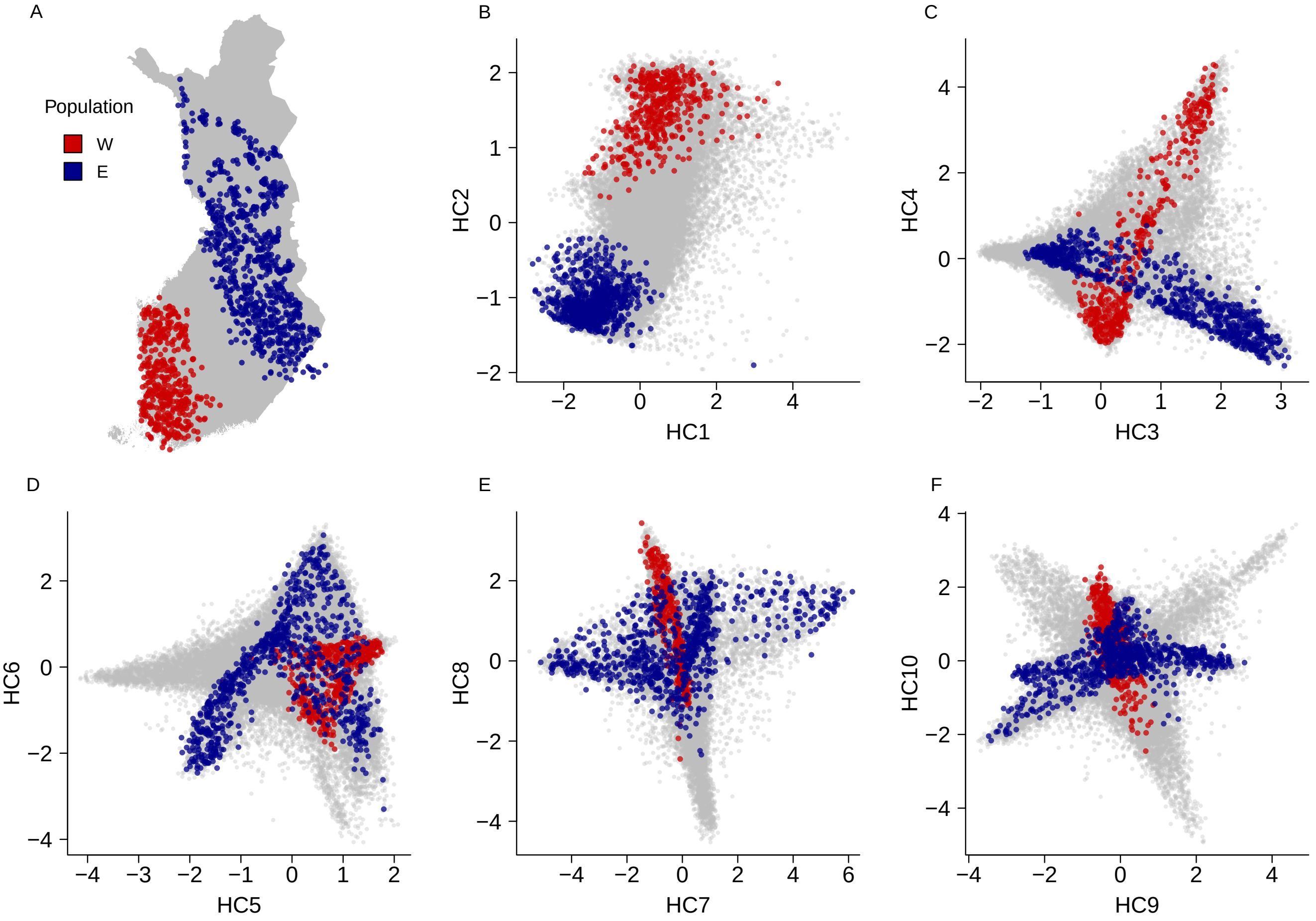

Supplementary Figure S16: A) Map of Finland with the geographical locations of the reference subset samples (n = 1,266) allocated to 2 Finnish source populations. The points depict the mean coordinates of the parents' municipalities of birth. B-F) The same individuals highlighted on the first ten haplotype components (HCs) from PBWTpaint. The samples not included in the reference groups are depicted in grey colour. W: West, E: East.

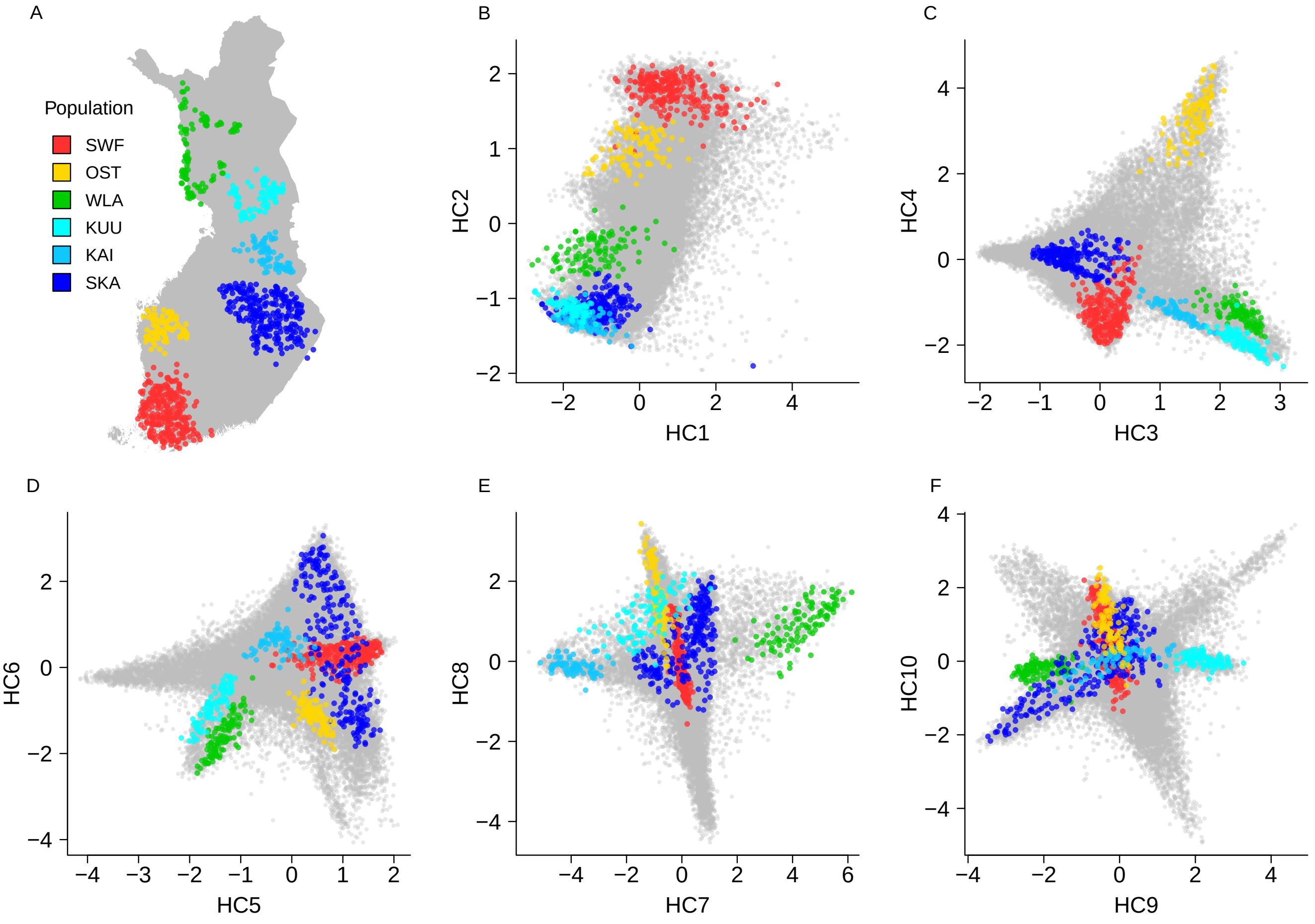

Supplementary Figure S17: A) Map of Finland with the geographical locations of the reference subset samples (n = 882) allocated to 6 Finnish source populations. The points depict the mean coordinates of the parents' municipalities of birth. B-F) The same individuals highlighted on the first ten haplotype components (HCs) from PBWTpaint. The samples not included in the reference groups are depicted in grey colour. SWF: Southwestern Finland, OST: Ostrobothnia, WLA: West Lapland, KUU: Kuusamo, KAI: Kainuu, SKA: Savo-Karelia.

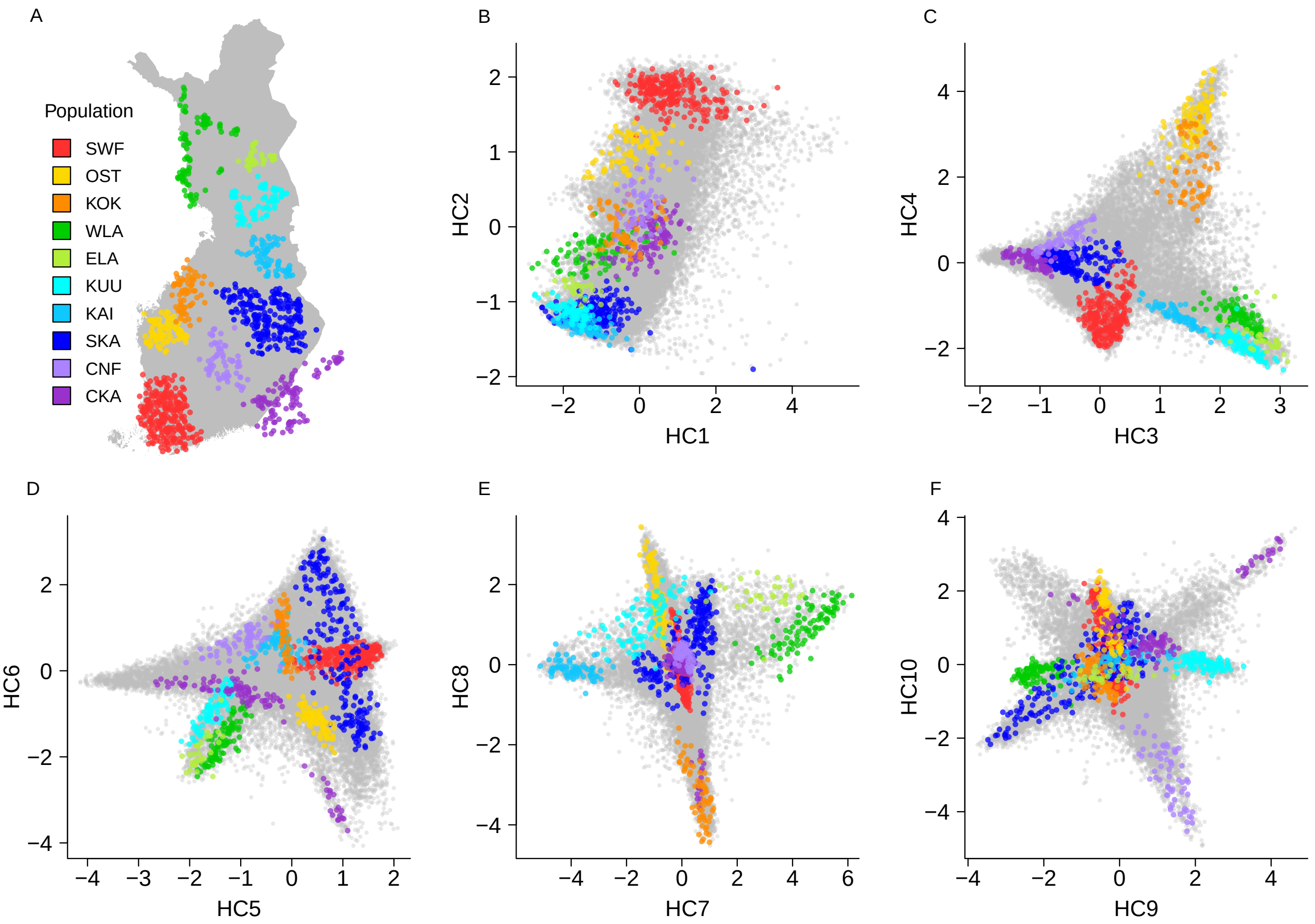

Supplementary Figure S18: A) Map of Finland with the geographical locations of the reference subset samples (n = 1,085) allocated to 10 Finnish source populations. The points depict the mean coordinates of the parents' municipalities of birth. B-F) The same individuals highlighted on the first ten haplotype components (HCs) from PBWTpaint. The samples not included in the reference groups are depicted in grey colour. SWF: Southwestern Finland, OST: Ostrobothnia, KOK: Kokkola, WLA: West Lapland, ELA: East Lapland, KUU: Kuusamo, KAI: Kainuu, SKA: Savo-Karelia, CNF: Central Finland, CKA: Ceded Karelia.

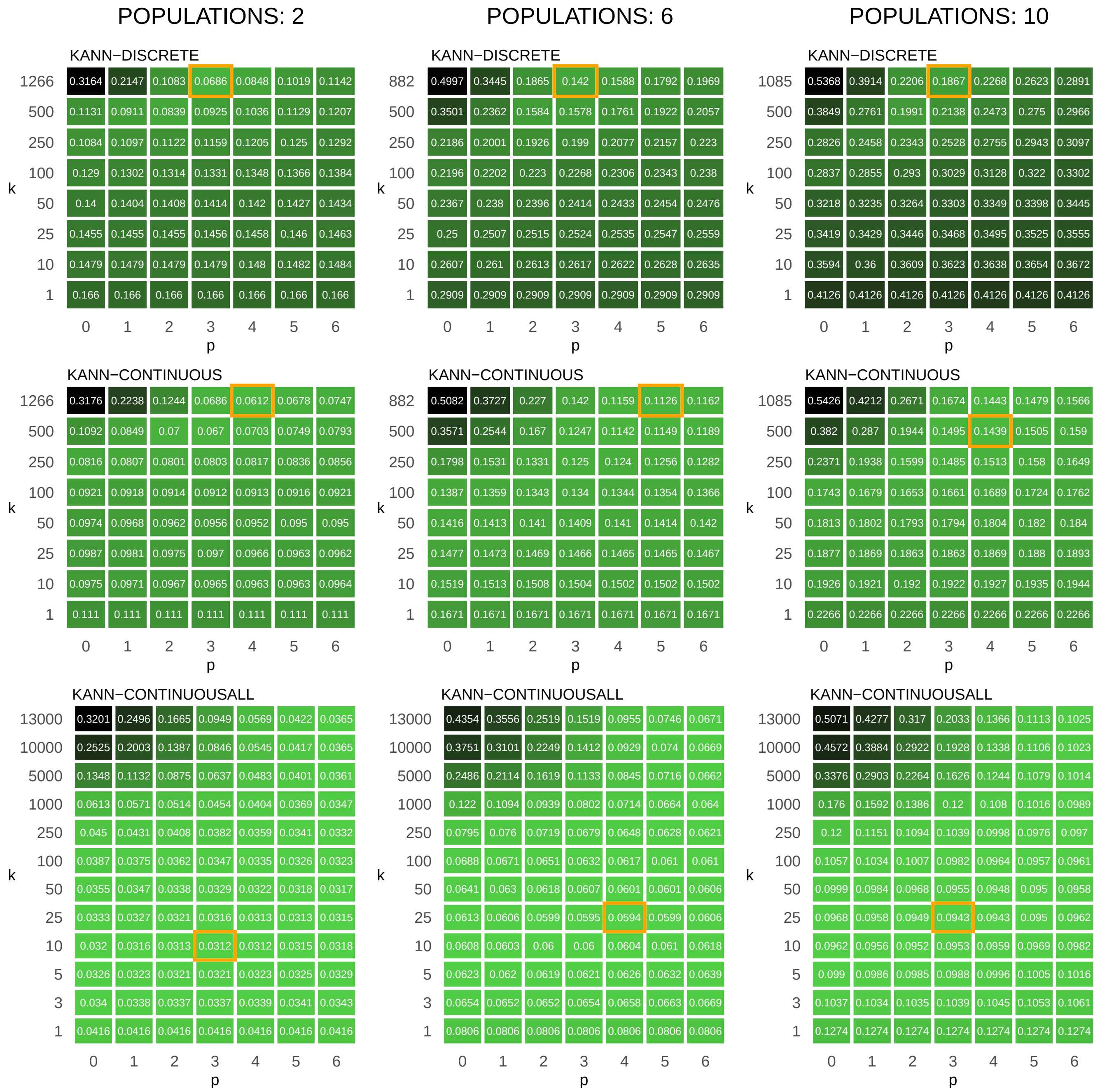

Supplementary Figure S19: Mean TVD of the query sample subset profiles estimated using KANN with the first 10 haplotype components and all parameter configurations considered. For the parameter p, values are shown from 0 to 6, since we did not observe any optimal parameter pairs with larger values of p when using the first 10 HCs. The TVD is shown for three optimization scenarios (DISCRETE, CONTINUOUS, CONTINUOUSALL) and with different numbers of source populations (2, 6, 10). The values are rounded up to four decimals and the minimum TVD is highlighted.

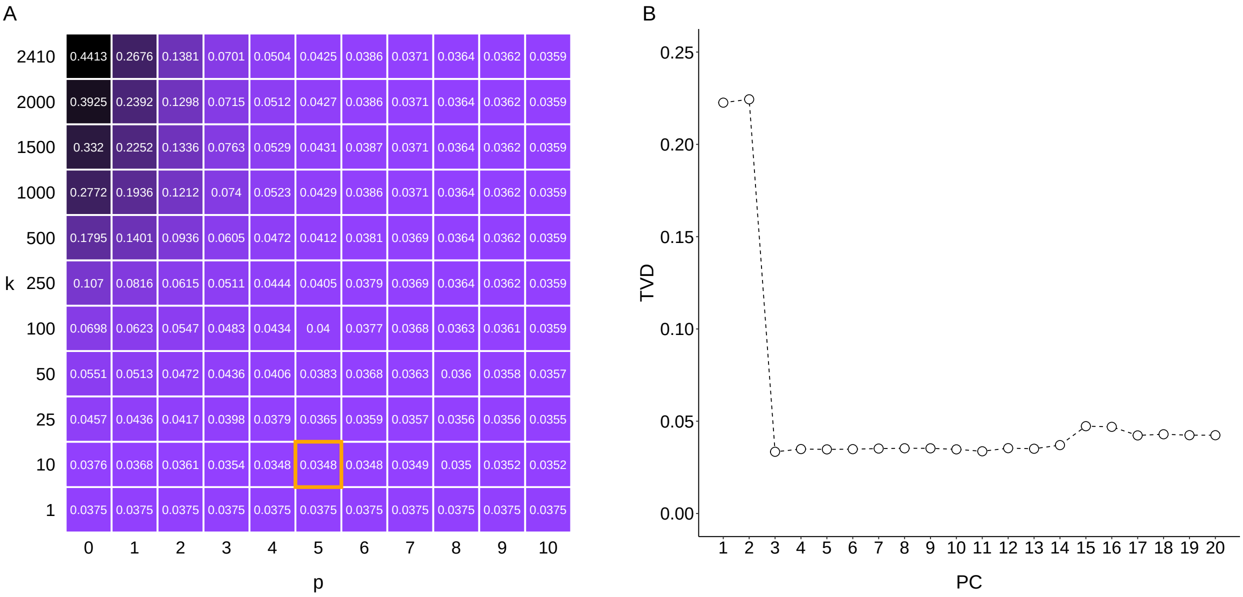

Supplementary Figure S20: A) Mean TVD of the 1KGP query sample set profiles estimated using KANN with all parameter configurations considered, and the first 10 PCs. The values are rounded up to four decimals and the minimum TVD is highlighted. B) The minimum mean TVDs (y-axis) obtained in the 1KGP query sample set across the parameter optimization process repeated with different number of principal components (x-axis).

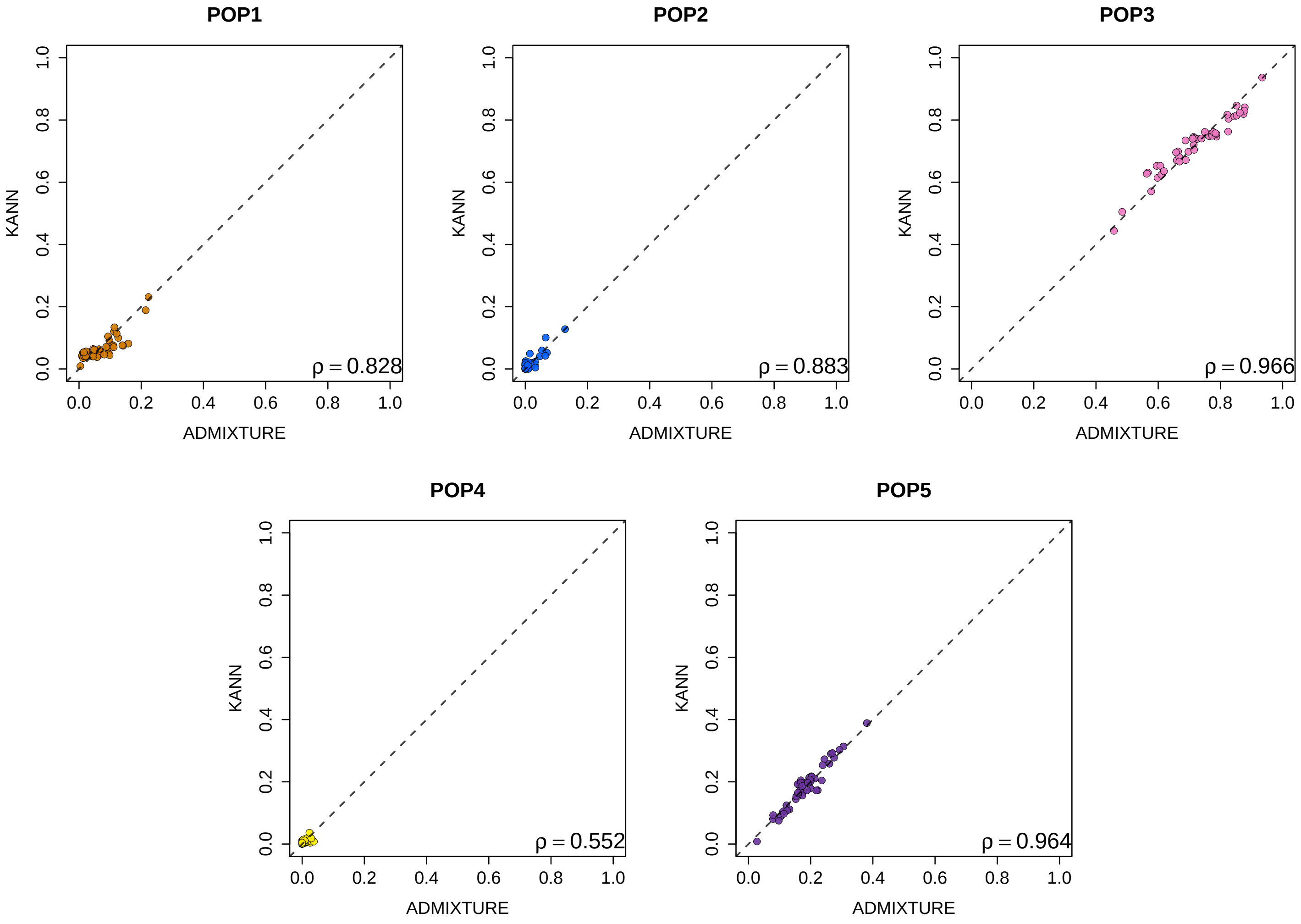

Supplementary Figure S21: Pairwise scatterplots of the 1KGP CLM test set samples' ancestry components estimated using KANN and ADMIXTURE for 5 populations. Diagonal is shown as a dashed line. Each panel reports the Pearson correlation coefficient (ρ) between the estimates of the two methods.
